# 5′ terminal nucleotide determines the immunogenicity of IVT RNAs

**DOI:** 10.1093/nar/gkae1252

**Published:** 2024-12-19

**Authors:** Magdalena Wolczyk, Jacek Szymanski, Ivan Trus, Zara Naz, Tola Tame, Agnieszka Bolembach, Nila Roy Choudhury, Karolina Kasztelan, Juri Rappsilber, Andrzej Dziembowski, Gracjan Michlewski

**Affiliations:** International Institute of Molecular and Cell Biology in Warsaw, Ksiecia Trojdena 4, 02-109 Warsaw, Poland; International Institute of Molecular and Cell Biology in Warsaw, Ksiecia Trojdena 4, 02-109 Warsaw, Poland; International Institute of Molecular and Cell Biology in Warsaw, Ksiecia Trojdena 4, 02-109 Warsaw, Poland; International Institute of Molecular and Cell Biology in Warsaw, Ksiecia Trojdena 4, 02-109 Warsaw, Poland; International Institute of Molecular and Cell Biology in Warsaw, Ksiecia Trojdena 4, 02-109 Warsaw, Poland; International Institute of Molecular and Cell Biology in Warsaw, Ksiecia Trojdena 4, 02-109 Warsaw, Poland; International Institute of Molecular and Cell Biology in Warsaw, Ksiecia Trojdena 4, 02-109 Warsaw, Poland; MRC Human Genetics Unit, Institute of Genetics and Cancer, University of Edinburgh, Western General Hospital, Crewe Road South, EH4 1QY Edinburgh, UK; International Institute of Molecular and Cell Biology in Warsaw, Ksiecia Trojdena 4, 02-109 Warsaw, Poland; Institute of Biotechnology, Technische Universität Berlin, Gustav-Meyer-Allee 25, 13355 Berlin, Germany; International Institute of Molecular and Cell Biology in Warsaw, Ksiecia Trojdena 4, 02-109 Warsaw, Poland; International Institute of Molecular and Cell Biology in Warsaw, Ksiecia Trojdena 4, 02-109 Warsaw, Poland

## Abstract

*In vitro* transcription (IVT) is a technology of vital importance that facilitated the production of mRNA therapeutics and drove numerous breakthroughs in RNA biology. T7 polymerase-produced RNAs can begin with either 5′-triphosphate guanosine (5′-pppG) or 5′-triphosphate adenosine (5′-pppA), generating potential agonists for the RIG-I/type I interferon response. While it is established that IVT can yield highly immunogenic double-stranded RNA (dsRNA) via promoterless transcription, the specific contribution of initiating nucleosides to this process has not been previously reported. Our study shows that IVT-derived RNAs containing 5′-pppA are significantly more immunogenic compared with their 5′-pppG counterparts. We observed heightened levels of dsRNAs triggered by IVT with 5′-pppA RNA, activating the RIG-I signaling pathway in cultured cells, as well as in *ex vivo* and *in vivo* mouse models, where the IFN-β gene was substituted with the mKate2 fluorescent reporter. Elevated levels of dsRNA were found in both short and long 5′-pppA RNAs, including those of COVID-19 vaccines. These findings reveal the unexpected source of IVT RNA immunogenicity, offering valuable insights for both academic research and future medical applications of this technology.

## Graphical Abstract

**Graphical Abstract:**
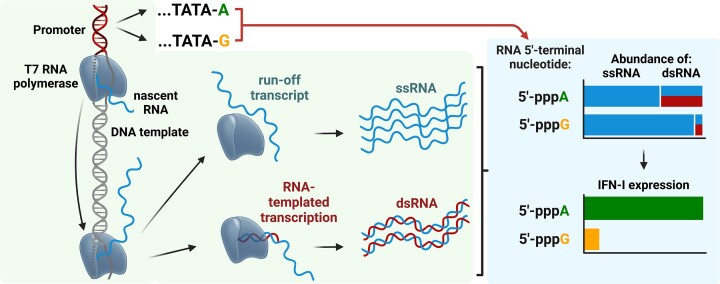

## Introduction

The innate immune system of higher eukaryotes is the first line of defense against invading pathogens, including viruses. Pathogen-Associated Molecular Patterns (PAMPs), which are recognized by cellular Pattern Recognition Receptors (PRRs), are shared between viral as well as endogenous RNAs. The 5′-triphosphate (5′-ppp) moiety is an important PAMP, present in many viral RNAs and endogenous polymerase III (Pol III) transcripts (1,2). Highly structured RNAs with 5′-ppp bind to Retinoic Acid Inducible Gene I (RIG-I/DDX58) PRR, provoking its conformational change and K63 ubiquitination by E3 ubiquitin ligase RIPLET (3,4), triggering a signaling cascade that culminates in phosphorylation of IRF3, IRF7 and NF-κB transcription factors (5). Upon activation, transcription factors translocate into the nucleus, initiating the expression of type I interferon (IFN), which in turn triggers an innate immune response. This response involves the upregulation of numerous interferon-stimulated genes (ISGs) (6) and is crucial for antiviral activity, but when overstimulated can lead to inflammation and autoimmune diseases (7).

The most potent activators of RIG-I are short, blunt, double-stranded RNAs (dsRNAs) capped with 5′-ppp or 5′-pp moieties (8–11). The 5′-terminal nucleotide must remain unmethylated at its 2′-O position to be recognized by RIG-I (12). dsRNAs with short 3′ overhangs or bulge loops can also serve as potent RIG-I ligands (9). Synthetic dsRNAs, including RNA duplexes and stem loop RNAs, with as few as 10 base pairs, have been also shown to activate RIG-I (13,14). Finally, investigations into immunogenic RNAs during influenza A virus (IAV) infection have identified 5′-ppp single-stranded genomic RNAs (ssRNAs) (15) as well as 80-nucleotide long mini viral RNAs (16) as effective stimulators for the RIG-I/IFN pathway.

*In vitro* transcription (IVT) employing T7 polymerase serves various purposes, including RNA production for research, mRNA-based drugs and vaccines (17,18). However, it introduces a 5′-ppp moiety that can activate the RIG-I/IFN pathway (19) and IFN-induced proteins with tetratricopeptide repeats 1 and 5 (IFIT1 and IFIT5 proteins) (20–22). Notably, IVT can also generate unintended dsRNA byproducts from random priming of abortive transcripts (23), turn-around transcription of run-off transcripts (24) or via promoterless transcription from sense strand of RNA or antisense strand of template DNA (19,25–27). Cellular response to those dsRNAs can result in undesirable suppression of protein synthesis and cell death, which in turn can drastically reduce the efficacy of mRNA therapies. Such dsRNA byproducts require complex purification processes and strategies to make the IVT less immunogenic, including purification by reversed-phase HPLC (28), cellulose fibers-mediated removal of dsRNAs (29) or incorporation of N1-methylpseudouridine (1mΨ) (30,31).

There are several classes of T7 promoters (32). Class III promoters, the most used, favor the incorporation of guanosine (G), while class II promoters facilitate efficient initiation from adenosine (A) or adenosine-containing coenzymes (33). Crucially, the ability to integrate either 5′-pppG or 5′-pppA by T7 polymerase has been leveraged in the production of SARS-CoV-2 mRNA vaccines. Moderna incorporated Cap1 enzymatically on the 5′-pppG-containing IVT RNAs (33), while BioNTech employed co-transcriptional addition of an AG-trinucleotide Cap1 analog (CleanCap) (34). However, even the most advanced capping protocols leave a fraction of uncapped, immunogenic 5′-ppp RNA (35). Although widely utilized in medicine and research, the impact of different 5′ nucleosides on the immunogenicity and purity of IVT RNAs has not been investigated.

Here, we demonstrate that despite their similar structures, IVT RNAs originating from 5′-pppA exhibit significantly higher immunogenicity compared to those originating from 5′-pppG. This difference was observed across human and mouse cultured cells and was further validated in our novel mouse model, where the IFN-β gene was replaced with the mKate2 fluorescent reporter. The activation of innate immunity by 5′-pppA RNAs relied on both RIG-I and the presence of 5′-ppp. Unexpectedly, our findings revealed that IVT reactions initiated with 5′-pppA generate significantly greater levels of highly immunogenic, blunt-end dsRNAs, compared to their 5′-pppG counterparts. Elevated amounts of dsRNA were detected in short 5′-pppA RNAs and full-length mRNAs, including erythropoietin (EPO) mRNA and COVID-19 vaccine mRNA harbouring the complete sequence of the spike protein, where terminal adenosine was employed during IVT reaction. This has significant implications for understanding the immunogenicity of IVT RNAs, existing mRNA medicines and their future development.

## Materials and methods

### Production of RNA with IVT reaction

RNA transcripts representing both viral and host origin, were prepared using IVT reaction (Supplementary Table S1). RNA representing miniviral RNA (36) was derived by truncating 76 nucleotides from segment 8^th^ of the IAV genome (GenBank: NC_002020.1). Short non-coding Y5 RNA (84 nucleotides; GenBank: NR_001571.2) was representing the host RNA.

The transcription template was first amplified using the High-Fidelity Phusion DNA Polymerase (Thermo #F530L) and primers (Supplementary Table S2) appending T7 Class III (TAATACGACTCACTATA) or ϕ2.5 Class II (TAATACGACTCACTATT) promoter sequence to produce 5′-pppA RNA or 5′-pppG RNAs, respectively. (37) Subsequent IVT reaction producing 5′-triphosphorylated RNAs was performed with NxGen T7 RNA Polymerase (Biosearch Technologies #30223–1). Then RNA was precipitated with 3 M Sodium Acetate (pH 5.1) and 100% EtOH, washed with 100% EtOH and resuspended in UP water. The RNA in 2 × loading buffer (7 M urea, bromophenol blue, and xylene cyanol) was then run on a denaturing polyacrylamide gel (10% polyacrylamide, 7.5 M urea in 1 × TBE) for 2 h. RNA was stained with Stains-all (Sigma-Aldrich #E9379) and bands of a specific size corresponding to respective RNA were excised with individual scalpel blades. Then RNA was extracted (0.3 M Sodium Acetate pH 5.2; 0.5 mM EDTA; 0.1% SDS) and precipitated again. RNA was washed with 100% EtOH, resuspended in UP water and filtered on Spin-X Centrifuge Tube Filters (Costar #8160) to remove remaining gel residues. RNA concentrations were assessed with NanoDrop and adjusted to 50 ng/μl. RNA was aliquoted and stored at -80°C. Denaturing PAGE/urea gel electrophoresis was repeated to confirm that no extra bands were observed in the final RNA preparation. Dephosphorylated RNA was produced with FastAP enzyme (Thermo #EF0654) and cleaned using column purification (Invitek Invisorb Spin Virus RNA Mini Kit). The 3p-hpRNA at a concentration of 100 ng/ml (InvivoGen #tlrl-hprna) or no RNA were used as positive and negative controls, respectively.

### Splint ligation

RNA was produced by ligation of *in vitro* transcribed 5′ acceptor RNA fragment with chemically produced 3′ donor RNA containing 5′ monophosphate essential for ligase activity using T4 Ligase 2 (NEB #M0239S). Additionally, the ssDNA fragment complementary to the 3′ end of the acceptor and the 5′ end of the donor was used as a splint to increase the specificity of ligation. Ligation was performed according to the manufacturer protocol for the T4 Ligase 2 enzyme. Ligated RNA was precipitated and purified from PAGE/urea gel with the procedure described in the IVT reaction section.

### Production of dsRNA

Fully complementary RNA strands were produced with IVT or splint ligation reaction described above. The sequences and primers are listed in the Supplementary Tables S1 and S2. Equal quantities of sense and antisense strands were mixed in a refolding buffer (0.1 M Tris-HCl pH 7.5, 0.1 M NaCl, 5 mM MgCl_2_) and incubated at + 80°C for 10 min and then at room temperature for 30 min. dsRNA was placed in tubes on ice and then used for further experiments.

### Native polyacrylamide gel electrophoresis

For native polyacrylamide gel electrophoresis, a native gel loading buffer (1 mM Tris-HCl pH 7.5, 5% glycerol, 0.001% bromophenol blue) was added to the samples and mixed. The mixtures were overlaid on a non-denaturing 12% polyacrylamide gel (prerun step was performed at 8 W for 45 min at + 4°C). After electrophoresis at 8 W for 120 min at + 4°C, the gels were stained with SYBR Gold nucleic acid gel stain (Invitrogen #S11494). Imaging was performed with a Chemidoc MP (Bio-Rad Laboratories).

### Cell culture

Murine bone marrow cells and fibroblasts were obtained from C57BL6J/Rj mice as previously described (38,39). Bone marrow cells were cultured with 20 ng/ml of macrophage colony-stimulating factor (BioLegend #576404) for 7 days as previously described (39) to generate BMDMs. Human A549, HEK293, THP-1, Calu1, HEK-Blue IFN-α/β cells, and murine MEF, BMDM, primary fibroblasts, B16-Blue IFN-α/β cells were maintained in Dulbecco's Modified Eagle's Medium (DMEM; Gibco #32430) supplemented with 10% fetal bovine serum (Gibco #10270–106). HEK-Blue IFN-α/β were cultured with 100 μg/ml Zeocine (InvivoGen #ant-zn-1) and 30 μg/ml Blasticidine (InvivoGen #ant-bl-05). B16-Blue IFN-α/β cells were supplemented with 100 μg/ml of Zeocine only. Primary cell cultures (fibroblasts and BMDMs) were established in presence of 1 × Penicillin-Streptomycin (Gibco #15140122) and 0.1 mg/ml Gentamicin (Gibco #15710064). Cells were cultured at + 37°C in a 5% CO_2_ humidified incubator. The list of cell lines used is provided in Supplementary Table S3.

### Transfection of RNAs in cell culture

Cells were seeded in a 12-well plate at a concentration of 0.3–0.7 × 10^6^ cells per well and incubated for 24–48 h. RNA was mixed with Lipofectamine: (100 ng RNA was prediluted in 125 μl of OptiMEM (Gibco #11058), then 2 μl of Lipofectamine 2000 (Invitrogen #11668) prediluted in 125 μl of OptiMEM was added) and after incubation at RT for 30 min 750 μl of cell culture medium was added. Leading to a final concentration of 100 ng RNA per ml of medium. RNA with medium was added to cells and incubated for 8 h (A549) or 24 h (HEK293, THP-1, Calu1, MEF, BMDM, primary fibroblasts). Supernatants and cell lysates in Roeder D Buffer (1.38 M glucose; 100 mM KCl, 2 mM EDTA; 100 mM Tris HCl pH 8; 0.2 mM PMSF and 12.5 mM DTT) were collected and processed either for IFN assay or western blot.

### Infection with IAV

Firstly, HEK293 cells were seeded in a 48-well plate at a density of 10^5^ cells per well in cell culture medium (DMEM supplemented with 10% heat-inactivated FBS). Secondly, after 24h of incubation (5% CO_2_, 37°C), cells were transfected with 100 ng/ml of RNA representing a fragment of the IAV genome (short viral RNA) in 0.4 ml per well. Thirdly, after another 24 h of incubation (5% CO_2_, 37°C), cells were washed with DMEM and inoculated with the NS1 mutant of the IAV (A/PR/8/34_NS1(R38A/K41A)) at an MOI of 0.0001 in 0.2 ml of medium (DMEM supplemented with 0.14% BSA). After 0.5 h of incubation (5% CO_2_, 37°C), one ml of virus growth medium (DMEM supplemented with 0.14% BSA and 0.2 μg/ml TPCK-treated trypsin) was added, and cells were incubated with the virus (5% CO_2_, 37°C). Around 50 μl of supernatant was collected at 0, 24, 48, 72 and 96 h and titrated with the endpoint dilution assay. Fifty percent cell culture infective dose (CCID50) endpoint titers were calculated by the Spearman–Kärber formula and expressed as a decimal logarithm.

### HEK-blue type I IFN assay

Supernatants from murine cells (MEF, BMDM, primary fibroblasts) were processed with B16-Blue and from human (A549, HEK293, THP-1, Calu-1) with HEK-Blue cells-based assays in quadruplicates. Twenty μl of supernatants (undiluted or prediluted 1:7) were added to 50 000 of HEK-Blue or 100 000 of B16-Blue cells in a 96-well plate. A standard curve was generated in parallel by serial dilutions of recombinant IFN-β standard in DMEM (R&D Systems #8499-IF-010 and #8234-MB-010). After overnight incubation (24 h), 20 μl of supernatants were mixed with 180 μl of the working solution of the QUANTI-Blue reagent (InvivoGen #rep-qbs) and incubated at 37°C for 0.1–3 h. Absorbance was measured at 654 nm using a Tecan's Sunrise absorbance microplate reader. Blank values were subtracted from all wells and the 4PL standard curves were fitted to provide semiquantitative analyses of the IFN concentrations produced in the RNA-transfected cells (40,41).

For estimating the apparent dissociation constant (K_d_), first, the optical density values for the blank wells were subtracted from the experimental data. In the colour reaction controlled by alkaline phosphatase, the optical density increased at higher RNA concentrations, and the data were fitted to a four-parameter (4PL) sigmoid curve model (OpticalDensity = Bottom + (Top - Bottom) / (1 + (K_d_ / RNAConcentration)^Slope^) with one constraint applied (Bottom = 0). To identify outliers in the dataset, Robust regression and OUTlier removal algorithm (ROUT) was employed. The false discovery rate was set at a 1% level (Q = 1%) (42). To compare K_d_ values between different datasets, we applied the entropy maximization principle using Akaike's Information Criterion corrected for small sample size (AICc). The probability of a shared K_d_ value fitting both models was computed using the relative likelihood (Akaike's Probability = 1 - (e^ΔAICc / 2^ + 1)^−1^).

### Detection of dsRNAs in IVT-derived RNAs using dot blot analysis

RNAs were produced with IVT reaction and fully complementary strands were mixed in equal quantities to produce dsRNAs as described above. Refolded RNA was diluted to contain 2 μg, 200 ng, 20 ng, 2 ng or 0.2 ng in 5 μl for dot blotting. DNase I treated RNA was produced according to the protocol provided (Thermo # EN0521) and cleaned using column purification. RNA solution was spotted on a positively charged nylon membrane (Invitrogen #AM10100) and each dot had an equal volume of 5 μl. The dots were air-dried and then crosslinked to the membrane using 120 mJ/cm2 UV (254 nm) light for 30 s. Membranes were briefly washed with PBS (Gibco #14 190) containing 0.1% Tween (Sigma-Aldrich #P9416). Membranes were then blocked for 2 h at room temperature with blocking solution that contained 50 μg/ml Sheared Salmon DNA (Thermo #AM9680), 5% non-fat milk wt:vol and 0.1 μl/ml RiboLock (Thermo #EO0381) in PBS-Tween. The blocked membranes were incubated with J2 antibody (Jena bioscience #RNT-SCI-10010200; 1/1000) diluted in PBS-Tween with 2% non-fat milk wt:vol overnight at + 4°C. Then, the membranes were washed three times with PBS-Tween, incubated with secondary antibodies (Polyclonal Goat Anti-Mouse Immunoglobulins/HRP, Agilent Dako #P0447, 1/2000) diluted in PBS-Tween with 2% non-fat milk wt:vol for 1 h at room temperature and washed three times with PBS-Tween. For the chemiluminescence reaction, peroxide ECL reagents (Bio-Rad #170–5061) were applied and then, the membranes were visualized using a Chemidoc MP (Bio-Rad Laboratories).

RNAs from Moderna and BioNTech SARS-CoV-2 vaccines were isolated by TRIzol™LS Reagent extraction (Invitrogen #10296028) according to the manufacturer's instructions and dissolved in nuclease-free water. The RNA utilized to generate the standard curve of the amount of dsRNA in SARS-CoV-2 vaccines RNAs was the dsRNA genome of bacteriophage Φ6 (kind gift from dr Krzysztof Skowronek, IIMCB). Φ6 dsRNA was diluted to contain 500, 250, 125, 63, 31 and 16 pg in 5 μl for dot blotting. SARS-CoV-2 vaccines RNAs were diluted to contain 400, 200, 100, 50, 25 and 13 ng in 5 μl for dot blotting. The RNAs were then processed in the same way as IVT-derived RNAs described above.

Standard curve generated by image analysis densitometry of fully complementary RNA was used to calculate the level of dsRNA contamination in IVT-derived RNAs.

### dsRNA immuno-northern blot

Around 2 μg of ssRNAs or dsRNAs were subjected to non-denaturing polyacrylamide gel electrophoresis as described above. Then, RNA transfer to a positively charged nylon membrane was performed using a wet transfer apparatus. Cold 0.5 × TBE buffer was used as a transfer buffer. The RNA transferred to the membranes was then crosslinked to the membrane using 120 mJ/cm2 UV light for 30 s. Membranes were briefly washed with PBS-Tween and then blocked for 2 h at room temperature with blocking solution (50 μg/ml Sheared Salmon DNA, 5% non-fat milk wt:vol and 0.1 μl/ml RiboLock in PBS-Tween). The blocked membranes were incubated with J2 antibody (1/1000) diluted in PBS-Tween with 2% non-fat milk wt:vol overnight at + 4°C. Then, the membranes were washed three times with PBS-Tween, incubated with HRP-conjugated anti-mouse secondary antibody (1/2000) diluted in PBS-Tween with 2% non-fat milk wt:vol for 1 h at room temperature and washed three times with PBS-Tween. For the chemiluminescence reaction, peroxide ECL reagents were applied and then, the membranes were visualized using a Chemidoc MP.

### Western blotting

Cell monolayers were washed once with ice-cold 1 × DPBS and resuspended in 50 μl of a Roeder D buffer (100 mM KCl, 20% (p/v) glycerol, 0.2 mM EDTA, 100 mM Tris HCl pH 8.0, 0.5 mM DTT, 0.2 mM PMSF). After vortexing, cells were sonicated with Diagenode's Bioruptor Pico sonication device for 10 min and centrifuged (16 000 × g, 5 min, +4°C). Supernatants were moved to pre-chilled low protein binding tubes and after quantifying protein concentration with NanoDrop stored at -80°C. Twenty to hundred μg of protein extract was mixed with 5 × SB buffer (250 mM Tris-HCl, 10% SDS, 50% glycerol, 250 mM DTT, 0.02% bromophenol blue) and resolved using a 10% gel. Proteins were transferred to nitrocellulose membranes (Amersham #10600007) using a wet transfer apparatus. Membranes were blocked with 1% Western Blocking Reagent (WBR; Roche #11 921681001) or 5% BSA in TBST buffer for 1 h at room temperature. Membranes stained for pIRF3 were blocked with 5% BSA in TBST buffer. The blocked membranes were incubated with primary antibodies diluted in 0.5% WBR in TBST overnight at + 4°C (Supplementary Table S4), washed three times with TBST, incubated with secondary antibodies diluted in 0.5% WBR in TBST for 1 h at room temperature (Polyclonal Goat Anti-Rabbit Immunoglobulins/HRP, Agilent Dako #P0448, 1/2000), washed three times with TBST. For the chemiluminescence reaction, peroxide ECL reagents (Bio-Rad #170–5061) were applied to each band and then, the membranes were visualized using a Chemidoc MP (Bio-Rad Laboratories).

### SHAPE analysis

RNA was processed with the EclipseBio SHAPE Single RNA Kit according to manufacturer's instructions. Briefly, re-folded RNA molecules were probed with NAI (2-methylnicotinic acid imidazolide), a structure probing agent, or DMSO control. Then an adapter was ligated to the 3′ end of the RNA and during reverse transcription reaction NAI induced mutations in the produced cDNA. Later, PCR-amplified libraries were subjected to Illumina 100-bp single-ended sequencing. We processed two replicates for Y5 and three replicates for short viral RNAs. For each replicate of NAI and DMSO, the data underwent the following analysis: UMI extraction with *umi_tools*, quality, and adapter trimming with *cutadapt*, repetitive element removal, alignment to hg38 using the *STAR aligner*, and PCR deduplication with *umi_tools* (10 bp UMI sequence). The specific alignment parameters were like those used by ShapeMapper2 (43). To access coverage depth reads from DMSO-treated RNA samples were split into either negative or positive strands with *samtools* and analyzed with *bedtools*.

Next, mutations (mismatches, insertions, deletions) and coverages were counted with custom Python scripts to compute mutation rates per base. Finally, NAI and DMSO mutation rates were subtracted and scaled with the interquartile range, which is the raw reactivity divided by 1.5×(Q_3_-Q_1_). With Q_3_ and Q_1_ being the 75^th^ quartile and the 25^th^ quartile of the raw reactivities per RNA. Reactivities for all bases with > 10 000 × coverage was reported.

Statistical comparison between reactivity profiles of two RNAs was conducted via the empirical Bayes (eBayes) t-test implemented in the Bioconductor package *limma* (44). Reactivity scores per replicate were square-root transformed. Next, a standard linear model was fitted, and a t-test was performed to identify positions where both RNA molecules have significantly different reactivity scores. The eBayes method shrinks position-wise variances for the computation of t-statistics which effectively increases the degrees of freedom, and consequently, the power to identify significant differences. Finally, mean reactivity per RNA was plotted. *P* < 0.001 was considered statistically significant.

### IFN-β/mKate2 mouse

A genetically modified mouse line was created in the Genome Engineering Unit at the International Institute of Molecular and Cell Biology (https://geu.iimcb.gov.pl/).

The mKate2 fluorescent marker nucleotides sequence, optimized for the mouse genome was inserted at the beginning of the IFN-β coding sequence [GenBank: NM_010510.2] using the CRISPR/*Cas9*-based methodology as previously described (45) (Supplementary Figure S5). Briefly, C57BL6J/Rj zygotes were microinjected with *Cas9* mRNA (25 ng/μl), sgRNA (GGUAGCAGCCGACACCAGCC; 12.5 ng/μl), and repair template dsDNA with 60 bp homology arms. To ensure proper transcription termination, two SV40 early mRNA polyadenylation signal sequences were added after the mKate2 sequence. An additional G > C mutation in the 5′ UTR was introduced to destroy the PAM sequence. Zygotes were surgically transferred to surrogate females and pups were screened by the PCR method using primers flanking the inserted sequence (forward primer: TGGGAAATTCCTCTGAGGCAG; reverse primer: AGGCAGTGTAACTCTTCTGCATC) and the presence of the correct insert (Supplementary Figure S5) was confirmed through Sanger sequencing. Founder mice were backcrossed with wild-type mice to get N1 generation animals. For routine genotyping, DNA from ear tips was extracted using the HotShot method (46), and a 3-primer method was used for PCR (forward primer: TGGGAAATTCCTCTGAGGCAG; reverse primer #1: AGGCAGTGTAACTCTTCTGCATC; reverse primer #2: TTACCGGTGGCCGAGCTTAC).

Mice were bred and maintained in the animal facilities of the IIMCB under standard conditions with the approval of the Polish Ministry of the Environment (decision #29/2023). The housing conditions in ventilated cages (Tecniplast #EM500) filled with wood chip bedding, enriched with nesting material, plastic houses, and cardboard tubes were in adherence with the Regulation of the Minister of Agriculture and Rural Development of 29 April 2022.

Mice were provided *ad libitum* with water and rodent feed (Altromin #1324TPF). The environmental conditions included a relative humidity of 45–65% and a room temperature of 20–24°C. The ventilation system ensured 75 air changes per hour for each cage. The lighting schedule followed a 12-h light/12-h dark cycle (lights on from 6:00 to 18:00).

Regular health monitoring was conducted at the IDEXX laboratory, and all animal procedures adhered to the guidelines of the EU Directive #2010/63/EU for animal experiments.

### RNA encapsulation into lipid nanoparticles

Lipid mix composition (50:10:38.5:1.5 molar ratio of DLin-MC3-DMA:DSPC:Cholesterol:PEG2000-DMG) was composed as previously described (47). RNA was encapsulated with a vortex mixing method (3:1 vol:vol ratio of RNA to lipid) and the N/P ratio (cationic nitrogen groups from the ionizable lipid over anionic phosphate groups from the mRNA) was ∼3. Buffer was exchanged for 1 × DPBS and LNPs were concentrated by centrifugation on Amicon 50 kDa filter unit (Merck #UFC805024). Size distribution and polydispersity index were determined using dynamic light scattering on Zetasizer Ultra Red (Malvern) in PBS buffer at 25°C in backscatter mode. Encapsulation efficiency and concentration of mRNA entrapped in LNPs were determined using the Quant-iT Ribogreen RNA assay (Invitrogen #R11490) by comparing fluorescence intensities in the presence or absence of 0.1% (w/v) Triton X-100.

### IV injection of RNA-LNP complexes in IFN-β/mKate2 reporter mice and preparing cell suspensions

The study protocol (#WAW2/124/2023) was approved by the 2^nd^ Local Ethics Committee for Animal Experimentation in Warsaw. Randomly selected IFN-β/mKate2 heterozygous male animals over 6 weeks of age were intravenously injected with 100 μg/kg of RNA encapsulated into LNP-RNA complexes and resuspended in sterile 0.85% NaCl up to 60 μl. In 24 h, animals were euthanized with CO_2_ and exsanguinated. The spleen and liver were extracted, minced with scissors, and pressed through a 70 μm strainer (Corning #431751) in the presence of ice-cold HBSS (Gibco #14025092). Cells were centrifuged at 200 × g for 3 min at 4°C and the pellet was washed. Red blood cell depletion was made with lysis buffer (BioLegend #420301) according to manufacturer's recommendations.

### Flow cytometry

Flow cytometry was performed on a Beckman Coulter CytoFLEX flow cytometer. Data acquisition and analysis were done with Beckman Coulter CytExpert 2.3 software. Dead cells were excluded from the analysis using live/dead Fixable Violet Dead Cell Stain Kit (Life Technologies #L34955) according to the manufacturer's protocol. Life/dead stain was excited using a 405 nm laser and the signal was detected using a 450/45 detector. mKate2 was excited using a 561 nm laser and the signal was detected using a 610/20 detector. The compensation matrix for relevant channels was calculated by the software. For the 450/45 detector, the compensated value was 14.57 and for the 610/20 detector the compensated value was 1.4. One million events were collected per sample.

### Image acquisition and analysis with Opera Phenix

BMDM plated in 96‐well plates (Greiner Bio-One #655090) at a density of 10^4^ cells per well were subjected to lipofection with RNAs. Then nine non-overlapping images per well with a resolution of 1080 × 1080 were captured on an hourly base using a 10 × objective and 2 × binning in non-confocal mode on the PerkinElmer Opera Phenix High-Content Screening System. Image acquisition and quantitative analysis were performed using the built-in software, Harmony 4.9. The chamber temperature was maintained at 37°C, with CO_2_ levels set at 5%.

Before analysis, flat-field correction was applied and, subsequently, cells were segmented based on the mKate2 signal. The subpopulation of cells with 150–800 μm^2^ of area occupied that started to exhibit mKate2+ ('+'-positive) signal with a mean intensity of ≥ 400 was counted.

### HEK293 RIG-I CRISPR/*Cas9* knockout and A549 MAVS CRISPR/*Cas9* knockout

HEK293 cells were co-transfected with 200 ng of GeneArt CRISPR Nuclease mRNA (Invitrogen #A29378) in addition to sgRNA prepared by mixing fluorescently labelled Alt-R CRISPR-*Cas9* tracrRNA, ATTO 488, (IDT #10010170) with Alt-R CRISPR-*Cas9* crRNA targeting sequence in intron 1 of DDX58 gene encoding RIG-I protein (IDT #Hs.Cas9.DDX58.1.AA – GGAUUAUAUCCGGAAGACCC) at 4 nM final concentration. After 24 h, fluorescence-positive cells were sorted using BD FACSAria II cell sorter to a 96-well plate at a seeding concentration of one cell per well, and the cells were grown in penicillin/streptomycin (Gibco #15140–122) containing DMEM until single colonies were established. Next, cells were split into two 96-well plates, one of which was used for a dot blot analysis. For the dot blot analysis, cells were washed once in cold PBS before adding 20 μl of Roeder D Buffer per well and sonication for 10 min (30 s ON/30 s OFF). Six microliters of protein from each well were spotted directly onto a nitrocellulose membrane followed by western blot. Selected clones were seeded from the second 96-well plate into 6-well plates and grown. The RIG-I levels were validated by standard western blot. Recombinant human IFN-β (R&D Systems #8499-IF-010) treatment was used to induce RIG-I expression and to correctly select clones with no RIG-I detectable. A549 MAVS knockout cell line was kindly provided by Prof. Tomasz Lipniacki. The cell line was generated using CRISPR-*Cas9* as described in (48).

### RNA pulldown mass spectrometry (RP-MS) analysis

RP-MS assay was based on a previously described RNA pulldown SILAC Mass Spectrometry method (49). 500 pmol of *in vitro* transcribed and PAGE purified RNA was treated with 100 mM Sodium Acetate and 5 mM sodium (meta)periodate in 200 μl of water and rotated for 1 h at room temperature in the dark. The RNA was precipitated by adding 600 μl of 100% ethanol and 15 μl of 3 M Sodium Acetate and incubating on dry ice for 30 min, followed by centrifugation at 16 000 × g, +4°C for 20 min. The RNA pellet was washed with 70% ethanol, followed by 5 min centrifugation at 16 000 × g and resuspended in 500 μl of 100 mM Sodium Acetate pH 5.2.

For one reaction 250 μl of adipic acid dihydrazide-agarose beads (Sigma-Aldrich #A0802) were washed 3 × with 100 mM Sodium Acetate followed by centrifugation at 2000 × g, +4°C for 2 min, then mixed with 500 μl of the periodate oxidized RNA and incubated overnight at + 4°C in the dark with rotation. The RNA-beads were washed by mixing with 700 μl of 4 M KCl and rocking for 30 min at room temperature and centrifuged at 2000 × g for 5 min, then washed 2 × with 2 M KCl, 2 × with Buffer G (20 mM Tris-HCl pH 7.5, 137 mM NaCl, 1 mM EDTA, 1% Triton X-100, 10% glycerol, 1.5 mM MgCl_2_, 1 mM DTT and 200 μM PMSF) and 1 × with Roeder D followed by 2 min centrifugation at 2000 × g. Control beads with no RNA attached were also prepared.

One mg of total protein extract was added to RNA-beads. The mixture was supplemented with 1.5 M MgCl_2_, 25 mM creatine phosphate, 100 mM ATP, and 2.5 μl of RiboProtect Hu RNase Inhibitor (Blirt #RT35). The mixture volume was adjusted to 650 μl with nuclease-free water. The RNA-beads-cell lysates mixtures were incubated at 37°C for 30 min with shaking. After three washes with Buffer G, the beads were mixed with 60 μl of 5 × Sample Buffer. Proteins captured by RNA were denatured at 95°C for 10 min with shaking. Around 30 μl of the supernatant was loaded onto SDS-PAGE or NuPAGE gel and western blot or mass spectrometry analysis was performed to detect the proteins respectively.

For mass spectrometry analysis proteins were separated on gel (NuPAGE Novex 4–12% Bis-Tris gel, Life Technologies), in NuPAGE buffer (MOPS) for 10 min and visualized using InstantBlue stain (Abcam). The stained gel band was excised and de-stained with 50 mM ammonium bicarbonate (Sigma Aldrich) and 100% (v/v) acetonitrile (Sigma Aldrich) and proteins were digested with trypsin, as previously described (50). In brief, proteins were reduced in 10 mM dithiothreitol (Sigma Aldrich) for 30 min at 37°C and alkylated in 55 mM iodoacetamide (Sigma Aldrich) for 20 min at ambient temperature in the dark. They were then digested overnight at 37°C with 13 ng/μl trypsin (Pierce). Following digestion, samples were diluted with an equal volume of 0.1% TFA.

The equivalent of 50 ng of the digest was loaded on the Evotip™ using the standard producer protocol. We utilized EVOSEP coupled to a TimsTOF ULTRA (Bruker) mass spectrometer equipped with a Captive Spray II source (Bruker). The separation was carried out on a Performance Column OE measuring 8 cm × 150 μm ID, with a particle size of 1.5 μm, maintained at 40°C. We employed the standard 60SPD EVOSEP method, applying a 21-min gradient for a total sample-to-sample time of 24 min using Solvent A: 0.1% formic acid (FA) and Solvent B: acetonitrile (ACN)/0.1% FA (Thermo Fisher Scientific™ Optima LCMS grade).

The dia-PASEF acquisition scheme was optimized for a cycle time estimate of 1.38 s. The window scheme was designed to cover most of the charge 2 precursor ions in the range m/z 391 − 1142 and 1/K0 0.68 − 1.36, using 22 × 31 Th windows, with accumulation and ramp times of 100 ms. The mass spectrometer was operated in the ‘high sensitivity detection’ (‘low sample amount’) mode.

The DIA-NN software platform (51) version 1.9.1. was used to process the raw files from label-free DIA and the search was conducted against the *Homo sapiens* reference proteome UP000005640 (Uniprot, released in 2019). Precursor ion generation was based on the chosen protein database (automatically generated spectral library) with deep-learning based spectra, retention time and IMs prediction. Digestion mode was set to specific with trypsin allowing maximum of one missed cleavage. Carbamidomethylation of cysteine was set as fixed modification. Oxidation of methionine, and acetylation of the N-terminus were set as variable modifications. MS1 and MS2 mass accuracies were set to 15 ppm. The parameters for peptide length range, precursor charge range, precursor m/z range and fragment ion m/z range as well as other software parameters were used with their default values. The precursor FDR was set to 1%.

Protein intensities were quantified from peptide intensities with directLFQ Python package (52). Statistical analysis was performed with R [R Core Team (2021). R: A language and environment for statistical computing. R Foundation for Statistical Computing, Vienna, Austria. https://www.R-project.org/]. *Protti* R package (53) was used for quality control, data filtration, imputation of missing values and statistical significance calculation using a moderated t-test based on the *limma* R/Bioconductor package (44). For functional validation, *P*-values were adjusted for multiple testing with the Benjamini-Hochberg correction. Data visualization was performed using the *ggplot2* R package (54).

### IVT stability and 5′-end phosphorylation state analyses

HEK293 cells were transfected with short viral RNA and incubated for 24 h. After incubation total RNA was extracted with Trizol, DNA was removed using DNase I treatment and RNA was purified with GeneJET RNA Cleanup and Concentration Micro Kit (Thermo #K0841). Then, 1 mg of purified total RNA sample was treated in three different conditions – without enzyme, with XRN-1 (NEB #M0338S) alone, or with XRN-1 and RppH (NEB #M0356S) together. Reactions were performed at 37°C for 60 min in dedicated buffer for XRN-1 enzyme provided by the producer. Then, treated samples were processed with RNA clean-up again and prepared for quantification analysis by cDNA synthesis using microScript microRNA cDNA Synthesis Kit (Norgen Biotek #54 410). Quantities of short viral RNA upon treatment were measured with one-step qRT-PCR assay with β-actin mRNA as a reference RNA.

### Statistical analysis

All data are reported as mean ± standard deviation. Statistical analyses were performed using GraphPad Prism 10.2.1.

## Results

### Structures of viral and pol III 5′-pppA and 5′-pppG RNAs

**Figure 1.**
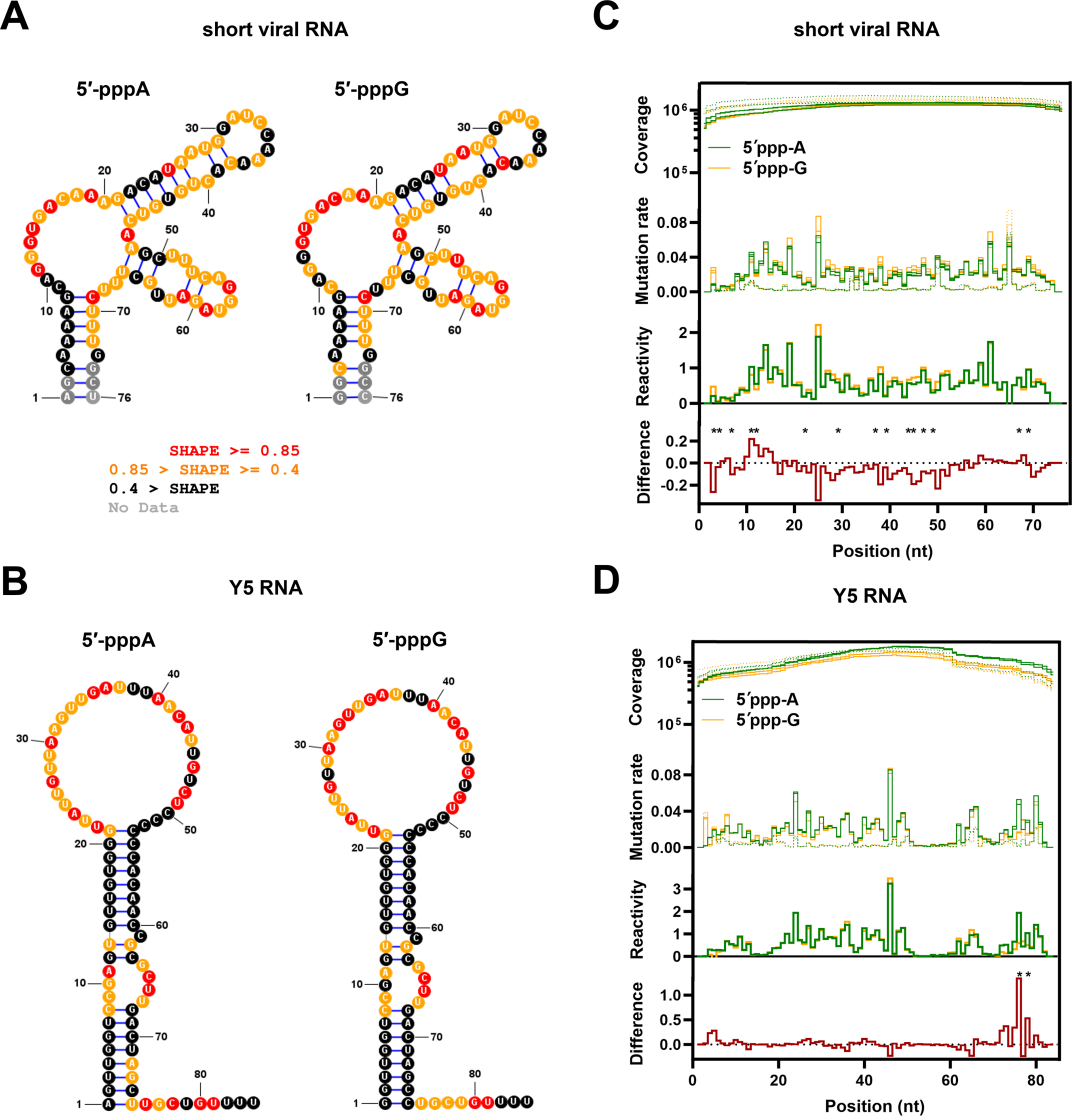
SHAPE analysis suggests no major structure rearrangements for short viral RNA and Y5 5′-pppA or 5′-pppG RNAs. (**A**, **B**) IVT RNAs starting either from 5′-pppA or 5′-pppG were subjected to SHAPE analysis. Secondary structures representing viral RNA and Y5 were predicted using the MFE algorithm with SHAPE constraints specified. (75) (**C**, **D**) Sequencing coverage exceeded the minimal threshold (10 000×) in all replicates of NAI-treated samples (solid lines) and DMSO-treated samples (dotted lines). The mutation rate was higher in NAI-treated samples (solid lines). Corresponding spikes in mutation, found in NAI and DMSO samples, might indicate a single-nucleotide polymorphism in the sample compared to the reference genome. All individual replicates (C: *n* = 3 for short viral RNA; D: *n* = 2 for Y5 RNA) are shown both in coverage and mutation line plots. The following part of the plot shows overlayed average reactivity levels between the set of RNAs. Higher SHAPE reactivity scores across the RNA of interest correlate with a higher likelihood of being unpaired. Negative reactivity values are excluded from the plot. The following part of the plot shows the difference between positive reactivity scores. Positions where reactivities are significantly different (*P* < 0.001) for A variant versus G variant comparison are marked with an asterisk (*).

To evaluate the significance of the 5′ terminal nucleotide identity derived from IVT, we initially employed two RNAs: IAV-derived short viral RNA and Pol III-derived Y5 RNA. The short viral RNA is a derivative of 3p-hpRNA (InvivoGen), a commonly used RIG-I ligand representing the first 87 nt of the positive strand of segment 8^th^ of the IAV PR8 strain genome. By truncating 11 nucleotides from the 3′ end, we created a 76 nt RNA that contains a blunt end panhandle structure, which should be an optimal RIG-I agonist and which we named *short viral RNA* (Figure 1A) (1,55). To see how the change of the 5′ terminal nucleotide affects Pol III transcripts, we have chosen Y5 RNA (Figure 1B), as it has been shown before to be endogenous trigger of the RIG-I/IFN pathway (56,57). To investigate the role of the terminal nucleotide, we replaced the initial 5′-pppA with 5′-pppG in both short viral and Y5 RNAs, while maintaining the base pairing between the 5′ and 3′-ends by creating Watson-Crick base pairs (A:U and G:C) for the initial 5′-pppA or 5′-pppG (Figure 1A and B). All RNAs, produced by IVT, were purified by denaturing preparative polyacrylamide/urea gel electrophoresis (PAGE) followed by cutting a specific band and then filtered to remove the remaining gel residues.

The RIG-I/IFN signaling pathway is highly dependent on RNA structure and any alterations in RNA ligand base pairing could have an inhibitory effect on the strength of the RIG-I/IFN response (58,59). To find out if the substitution of nucleosides at the RNA ends could affect the structure of 5′-pppA versus 5′-pppG RNAs, *in vitro* RNA Selective 2′-Hydroxyl Acylation analyzed by Primer Extension (SHAPE) analyses for both, short viral and Y5 RNAs, were performed. Secondary structure predictions using the maximum free energy (MFE) algorithm combined with SHAPE results revealed that all tested, cognate RNA pairs exhibited similar secondary structures (Figure 1A and B). This was supported by comparable SHAPE reactivity values for individual nucleotide positions between the 5′-pppA and 5′-pppG RNAs (Figure 1C and D). The Y5 RNA with 5′-pppA showed marginally more relaxed SHAPE reactivity in the lower portion of the stem when compared with the 5′-pppG RNA (Figure 1C and D). Short viral RNA pairs were confirmed to form blunt end panhandle structures, which contain 8-bp double-stranded fragments and one mismatch between the 4^th^ and 73^rd^ nucleotides (Figure 1A) (60). The Y5 RNA pair was found to form long, double-stranded fragments (containing one hairpin loop, single-base bulge, and asymmetric internal loop) with a 9-nt long 3′ overhang corresponding to polyuridine tail (Figure 1B), largely like what has been previously described (61).

### IVT 5′-pppA RNAs hyperstimulate the RIG-I/IFN pathway in human cells

**Figure 2.**
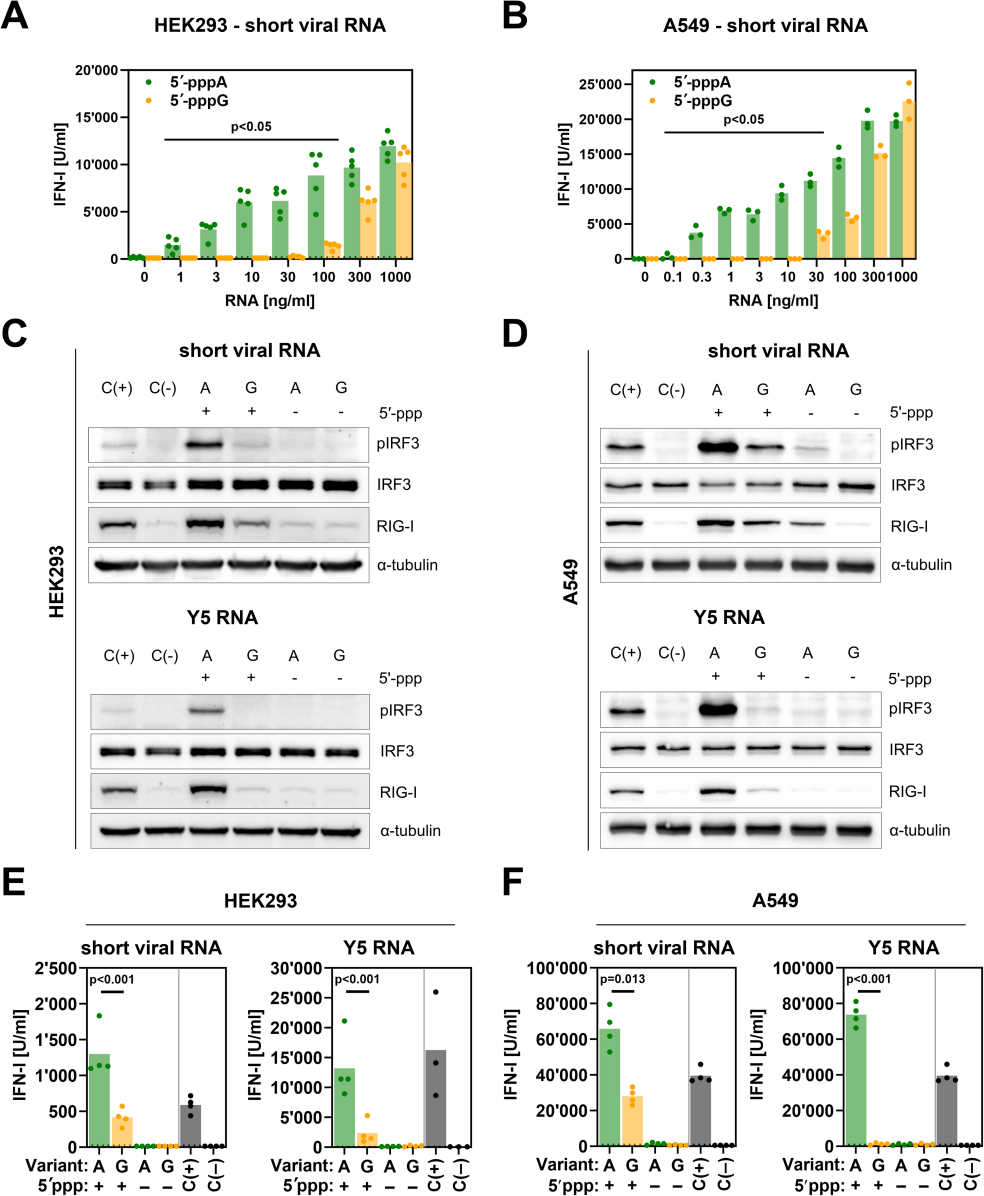
IVT 5′-pppA RNAs stimulate the RIG-I/IFN pathway in human cells more efficiently than 5′-pppG RNAs. (**A**) To compare the RIG-I/IFN responses triggered by RNAs that begin with 5′-pppA and 5′-pppG, IVT RNA, representing a fragment of the IAV genome, was transfected into HEK293 cells at various concentrations. The concentrations of type I IFN in the supernatants were assessed using the HEK-Blue IFN assay after 24 h. A selected concentration of RNA (100 ng/ml) was used to assess RIG-I levels and IRF3 phosphorylation with western blot upon treatment. (**C**) Dephosphorylated viral RNA (100 ng/ml) and Pol III transcript Y5 RNA were subjected to the HEK-Blue IFN assay. (**B**, **D**, **F**) Similar experiments were conducted in A549 cells, with type I IFN concentrations assessed in the supernatants and RIG-I levels and IRF3 phosphorylation in protein lysates in 8 h after treatment. The dotted line represents a lower quantification limit. The positive control (C(+)) involved transfection with 100 ng/ml 3p-hpRNA, while the negative control (C(−)) was mock-transfected with lipofectamine alone. (**A**, **B**, **E**, **F**) Upon log-transformation data were compared using two-way ANOVA with Šídák's multiple comparisons test. Experiments were conducted using either five (**A**), four (**E**, **F**) or three (**B**) biological replicates.

To elucidate the role of the 5′ terminal nucleotide present in IVT-derived RNAs on the strength of RIG-I/IFN pathway activation, we transfected IVT short viral RNAs harboring 5′-pppA or 5′-pppG as a 5′ terminal nucleotide into human embryonic kidney HEK293 and lung adenocarcinoma A549 cells. We used a wide range of RNA concentrations between 0.1 ng/ml and 1000 ng/ml. Using HEK-Blue IFN-α/β colorimetric assay, we assessed the expression of IFN-α/β that was stimulated by tested RNAs. Notably, we observed substantially more efficient activation of the RIG-I/IFN pathway by short viral RNA initiating with 5′-pppA compared to the one starting with 5′-pppG in both HEK293 and A549 cells (Figure 2A and B). The production of IFN-I increased several thousandfold when low concentrations of RNAs were used. Only at the highest RNA concentration (1000 ng/ml), no significant difference was observed between the 5′-pppA and 5′-pppG variants. Confirmatory findings were observed in the case of IRF3 protein phosphorylation, which was assessed with western blot analysis (Supplementary Figure S1A).

We compared the capability of IVT RNA to induce a type I IFN response by estimating the apparent dissociation constant (K_d_) for each RNA variant. There was a 92-fold difference in apparent K_d_ values when comparing 5′-pppA with 5′-pppG short viral RNAs in A549 cells, and a 17-fold difference in HEK293 cells (Supplementary Figure S1B and C). Cells exhibited significant IFN production and difference for 5′-pppA and 5′-pppG short viral RNAs as early as at 4 h into the treatment (Supplementary Figure S1D to G) reaching saturation after 24 h. Both types of RNAs (short viral RNA and Y5 RNA) initiating with 5′-pppA tested at a concentration of 100 ng/ml also proved much more potent in phosphorylating IRF3 and inducing RIG-I expression as confirmed by western blot analysis (Figure 2C and D, Supplementary Figure S1E and G), as well as elevating type I IFN, measured with HEK-Blue assay in HEK293 and A549 cells (Figure 2A, B, E and F, Supplementary Figure S1D and F). Moreover, the original 3p-hpRNA, which does not have a blunt end, also showed similar IRF3 phosphorylation pattern in both HEK293 and A549 cells when 5′-pppA and 5′-pppG variants were compared (Supplementary Figure S1H and I).

Control experiments demonstrated that RIG-I was crucial for the activation of the pathway by these and other tested RNAs (including 3p-hpRNA, EPO mRNA, and Seg 8^th^ PR8 RNA), as no efficient IRF3 phosphorylation was detected in RIG-I KO HEK293, A549 and THP-1 cells (Supplementary Figure S2A to C). Additionally, there were marginal levels of IRF3 phosphorylation and type I IFN production when dephosphorylated short viral and Y5 RNAs were used (Figure 2C–F). Transfection of RIG-I KO cells with poly I:C led to robust IRF3 phosphorylation, confirming the integrity of other innate immune sensing pathways (Supplementary Figure S2A–C). Furthermore, transfection of MAVS KO A549 and THP-1 cells with the tested RNAs resulted in no or marginal IRF3 phosphorylation (Supplementary Figure S2D and E). In contrast, MDA-5 KO A549 and THP-1 cells exhibited increased IRF3 phosphorylation in response to all tested RNAs (Supplementary Figure S2D and E). Differences in the immunogenicity of 5′-pppA and 5′-pppG short viral and Y5 IVT RNAs were observed in Calu-1 and THP-1 cell lines (Supplementary Figure S2F and G). Notably, pretreatment with 5′-pppA short viral RNA, unlike the 5′-pppG variant or dephosphorylated RNAs, significantly reduced IAV replication *in vitro* (Supplementary Figure S3). These findings suggest that IVT 5′-pppA RNAs activate the innate immune response via the RIG-I signaling pathway.

Next, to broaden our observation, we generated a structurally diverse collection of IVT 5′-pppA and 5′-pppG RNAs, predicted to form blunt ends or 3′ overhangs (the sequences and descriptions can be found in Supplementary Table S1). Irrespective of the predicted RNA structure, all tested 5′-pppA RNAs (excluding serine tRNA) exhibited much stronger RIG-I/IFN pathway stimulation observed as a higher IRF3 protein phosphorylation in HEK293 and A549 cells (Supplementary Figure S4A and B) and increased type I IFN production (Supplementary Figure S4C). These results show that IVT-derived RNAs initiating with 5′-pppA are much more immunogenic in human cells than cognate sequences starting with 5′-pppG and emphasize the significance of RNA structure, concentration and the duration of RIG-I/IFN pathway stimulation in this phenomenon.

### IVT-produced 5′-pppA RNAs hyperstimulate the RIG-I/IFN pathway in murine cells

**Figure 3.**
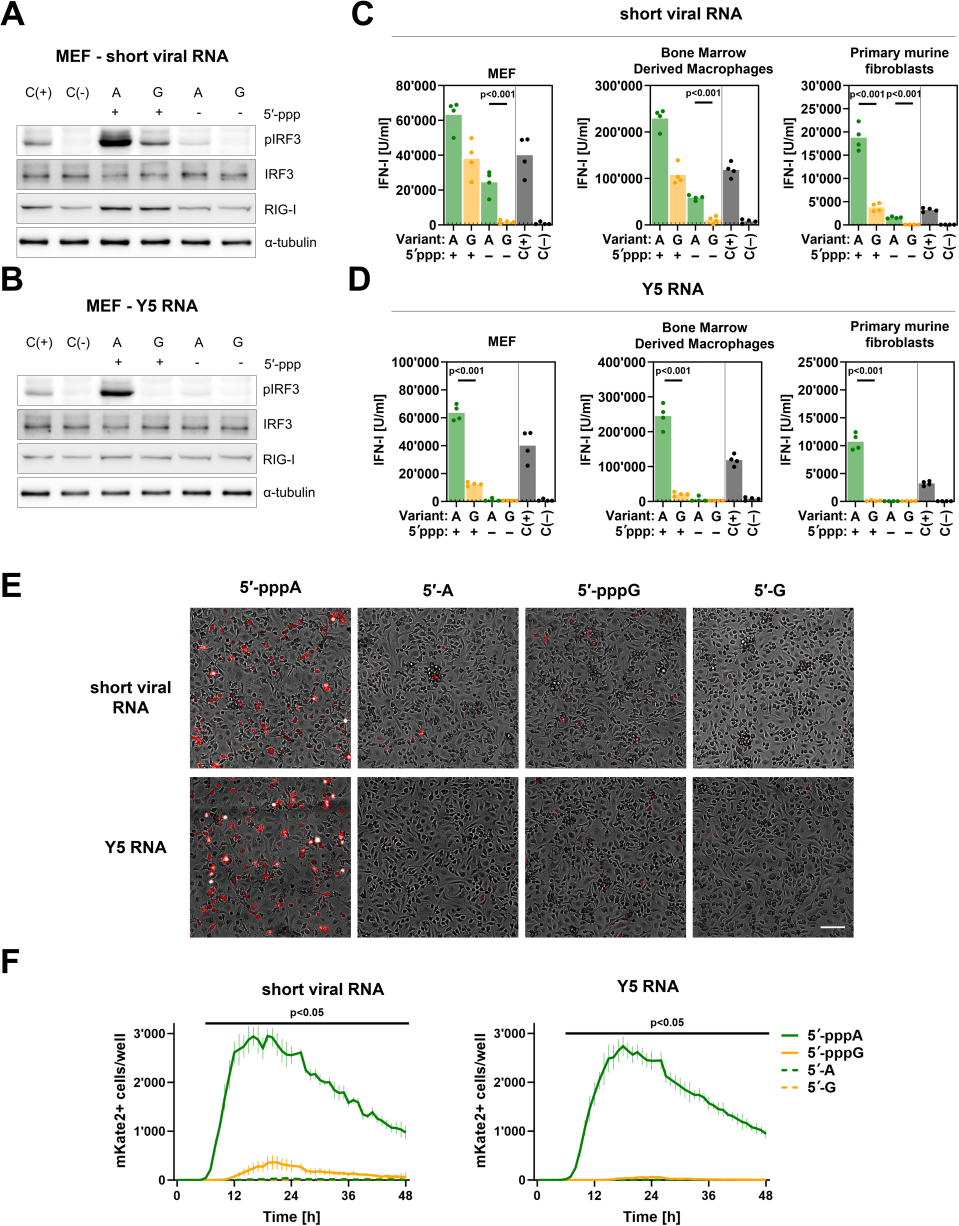
IVT 5′-pppA RNAs stimulate the RIG-I/IFN pathway in murine cells more efficiently than 5′-pppG RNAs. To compare the RIG-I/IFN responses triggered by RNAs that begin with 5′-pppA and 5′-pppG, IVT RNA, representing a fragment of the IAV genome, and Pol III transcript Y5 RNA were transfected into murine cells at a concentration of 100 ng/ml. (**A**, **B**) RIG-I levels and IRF3 phosphorylation in protein lysates were assessed using western blot. The positive control (C(+)) involved transfection with 100 ng/ml 3p-hpRNA, while the negative control (C(−)) was mock-transfected with lipofectamine alone. Upon log-transformation data were compared using two-way ANOVA with Šídák's multiple comparisons test. (**C**, **D**) The concentration of type I IFN in the supernatants was measured using HEK-Blue IFN assay at 24 h after treatment (*n* = 4). The dotted line represents a lower quantification limit. (**E**) Representative images of murine mKate+ BMDMs at 24 h upon RNA transfection. Scale bar is 100 μm. (**F**) Counts of mKate2+ cells at 0–48 h upon RNA transfection. Vertical lines represent standard deviation for *n* = 4. Longitudinal data upon shifted log-transformation (log_10_(x + 1)) were compared using repeated measures two-way ANOVA (rANOVA) with Geisser-Greenhouse correction and Šídák's multiple comparisons test.

To elucidate the impact of the 5′ terminal nucleotide type on the activation strength of the RIG-I/IFN pathway in murine cells, we repeated transfections with IVT RNAs in cell cultures of murine origin: murine embryonic fibroblasts cell line (MEF), primary cultures of bone marrow-derived macrophages (BMDMs) and skin-derived fibroblasts. Similarly, to human cells, western blot analysis revealed higher levels of phosphorylated IRF3 and increased RIG-I expression for RNAs starting with 5′-pppA compared to RNAs initiating with 5′-pppG (Figure 3A and B). Furthermore, IVT RNAs beginning with 5′-pppA triggered the type I IFN production to a significantly greater extent than those starting with 5′-pppG in all the tested cell cultures (Figure 3C and D). Upon dephosphorylation of the RNAs, we observed significantly reduced RIG-I/IFN pathway activation or type I IFN production (Figure 3A–D). Although some variation in sensitivity was observed across different cell cultures, noteworthy, the highest levels of type I IFN production were observed in immune BMDM cells (Figure 3C and D).

This observation prompted us to create a reporter knock-in mouse line in which the IFN-β gene was replaced with the mKate2 open reading frame (Supplementary Figure S5). By replacing the IFN-β gene with the reporter we aimed to limit the IFN feedback loop and toxicity, focusing mainly on the initial RIG-I/IFN pathway activation. We repeated the transfection experiments using reporter BMDM cells from homozygous mice, enabling us to monitor in real time IFN-β expression represented by mKate2 marker production (Figure 3E). Kinetic data from mKate2+ cells illustrate that the onset of IFN-β response starts as early as at 5 h post transfection with the response peaking at 20 h (Figure 3F). This response was much more potent for RNAs initiating with 5′-pppA and fully triphosphate-dependent. In summary, the elevated induction of the RIG-I/IFN pathway by 5′-pppA RNAs can be detected in human and mouse systems.

### IVT 5′-pppA RNAs hyperstimulate the RIG-I/IFN pathway *in vivo*

**Figure 4.**
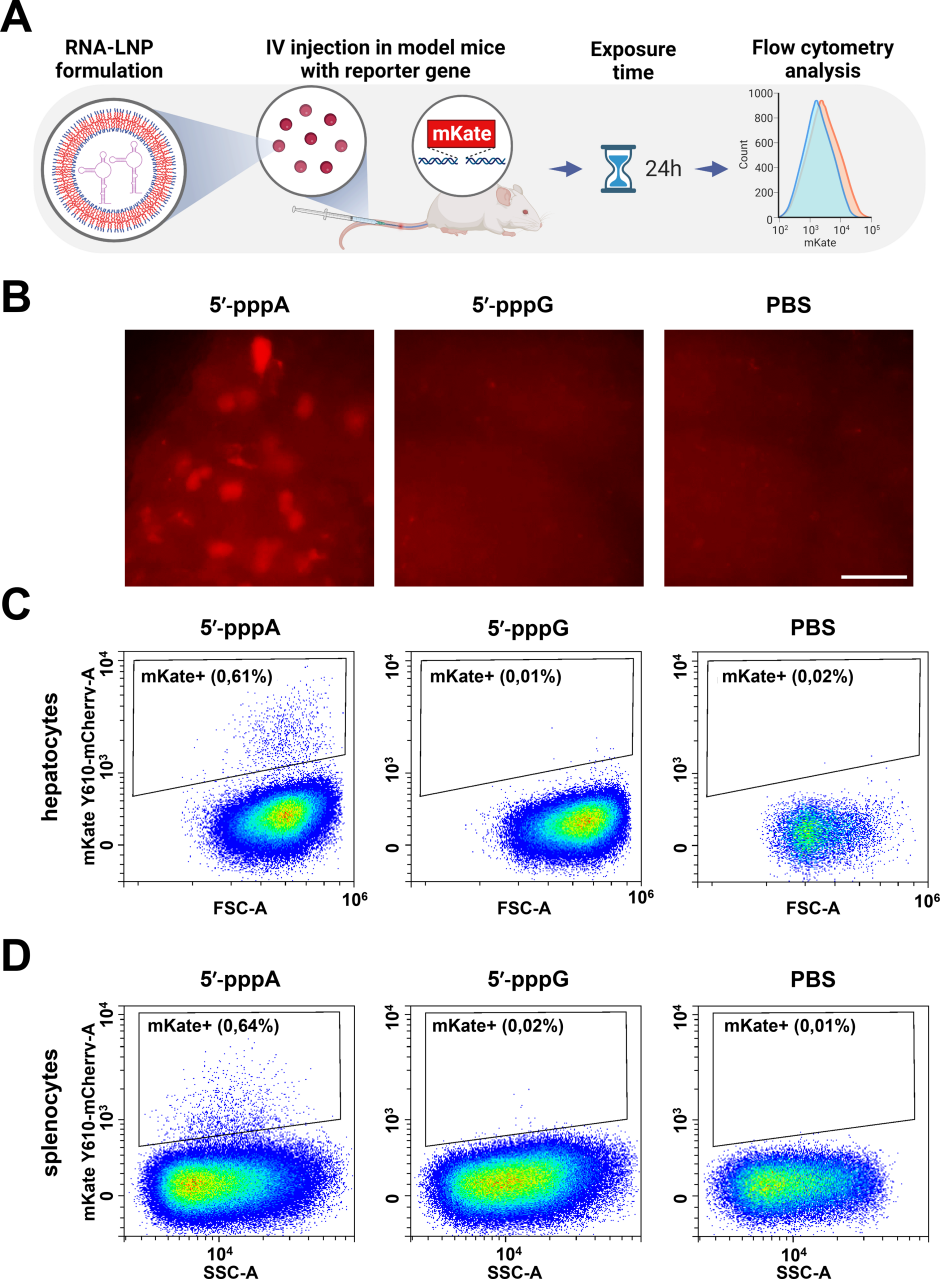
IVT 5′-pppA RNAs stimulate the RIG-I/IFN pathway in mice more efficiently than 5′-pppG RNAs. (**A**) Experimental setup: RNA was encapsulated into LNP and RNA-LNP complexes were IV injected (100 μg of encapsulated RNA per kg of body weight) in mice. Flow cytometry analysis of hepatocytes from reporter mice was conducted 24 h after RNA-LNP inoculation. (**B**) Fluorescent cells in liver. The injection of the 5′-pppA short viral RNA allowed the observation of fluorescent cells in liver before processing the organs into cell suspension. Scale bar is 50 μm. (**C**, **D**) Representative images for flow cytometry analysis of hepatocytes and splenocytes. (**C**) Liver: *P* = 0.0005 using one-way ANOVA with Šídák's multiple comparisons test; 5′pppA: M ± SD = 0.81 ± 0.17%, *n* = 3; 5′pppG: 0.11 ± 0.06%, *n* = 2; PBS control: 0.19 ± 0.12%; *n* = 5. (**D**) Spleen: *P* = 0.028 using one-way ANOVA with Šídák's multiple comparisons test; 5′pppA: M ± SD = 0.40 ± 0.29%, *n* = 3; 5′pppG: 0.01 ± 0.01%, *n* = 2; PBS control: 0.01 ± 0.01%; *n* = 5.

To test if the phenomenon of 5′-pppA RNAs hyperstimulation of the RIG-I/IFN pathway is observed at the organism level, we have injected the IVT RNAs encapsulated into lipid nanoparticles (RNA-LNP) in IFN-β/mKate2 reporter mice (Figure 4A). RNA-LNP complexes were injected intravenously (IV) into reporter mice within 48 h after production (Figure 4A). Twenty-four hours after administration, IFN-β/mKate2+ cells were observed both in the liver and spleen (Figure 4B to D). The number of gated cells with high level of mKate2+ signal in mice injected with 5′-pppA was 8 and 35 times higher compared to 5′-pppG RNA, as measured by flow cytometry in liver and spleen, respectively (Figure 4C and D). This shows that 5′-pppA RNA-mediated hyperstimulation of the RIG-I/IFN pathway can be also detected in the whole animal.

### IVT 5′-pppA RNAs contain a greater amount of dsRNAs compared to 5′-pppG RNAs

**Figure 5.**
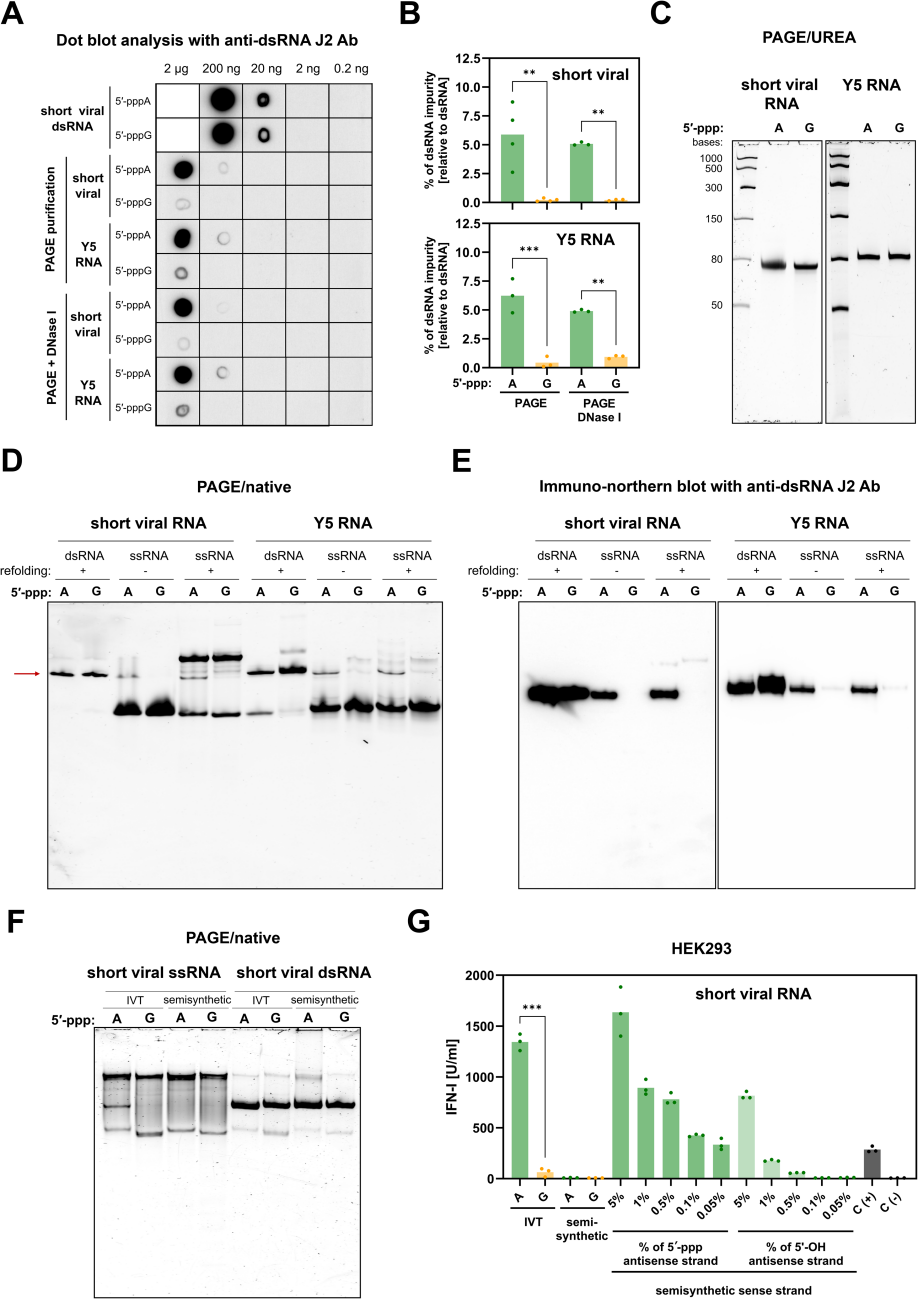
IVT-produced 5′-pppA RNAs contain more dsRNA compared with 5′-pppG RNAs. (**A**) Dot blot analysis of IVT short viral and Y5 RNAs was performed with anti-dsRNA J2 antibody, (**B**) then densitometric analysis (*n* = 4 for short viral RNA, *n* = 3 for Y5 RNA) was conducted to assess the level of dsRNA byproducts in IVT-derived RNAs. (**C**) PAGE/Urea analysis was done on short viral RNA and Y5 RNA. (**D**, **C**) PAGE in native conditions was conducted on IVT control dsRNAs (produced by annealing of sense and antisense RNA strands) and ssRNAs. (**E**) Immuno-northern blot using PAGE native gel followed by transfer to the membrane and detection with anti-dsRNA J2 antibody was used to detect dsRNA. (**F**) PAGE in native conditions of IVT short viral RNAs and semisynthetic short viral RNAs produced by splint ligation revealed the presence of dsRNA in IVT 5′-pppA RNAs but not in semisynthetic counterparts. (**G**) To assess the immunogenic potential of splint-ligated RNAs, an IVT-produced antisense RNA, with or without triphosphate moieties, was added to the sense splint-ligated RNA. The concentration of type I IFN in the supernatants was measured using HEK-Blue IFN assay at 24 h after treatment (*n* = 3). The positive control (C(+)) involved transfection with 100 ng/ml 3p-hpRNA, while the negative control (C(−)) was mock-transfected with lipofectamine alone. (**B**, **G**) Data were compared using two-way ANOVA with Šídák's multiple comparisons test.

To understand the mechanism behind the hyperstimulation of RIG-I/IFN pathway by IVT 5′-pppA RNA, we conducted a comprehensive assessment of the levels of dsRNA generated by IVT (19,25). For measurements of dsRNA impurities, we performed immunodetection using the anti-dsRNA J2 monoclonal antibody, which specifically recognizes dsRNA helices longer than 40 base pairs. We compared the IVT 5′-pppA and 5′-pppG RNA variants, including both short viral RNA and Y5 RNA. Dot blot analysis of RNA sample dilution series revealed approximately a 10-fold higher amounts of dsRNA in the 5′-pppA variants compared to the 5′-pppG variants both for short viral and Y5 RNAs (Figure 5A and B). As a positive control, we prepared dsRNA formed by mixing short viral RNA with its IVT fully complementary antisense strand in a 1:1 ratio. Our results suggest that the main source of enhanced immunogenicity of IVT-derived 5′-pppA RNAs is increased level of dsRNA production.

RNAseq performed while analysing SHAPE experiments revealed low proportion of reads mapping to the full length of the antisense strand (<0.1% for short viral RNA and < 0.4% for Y5 RNA) (Supplementary Figure S6A). Since our RNAs were purified using denaturing PAGE/Urea, we assumed that the differences in sequence coverage were due to sequencing bias. The ratios between sense and antisense strands were batch-dependent but generally higher for the 5′-pppA variants. Although trace amounts of residual contaminants, such as dsDNA from the original plasmid or PCR amplicon, were identified in the final RNA preparation, none of these contaminants triggered a type I IFN response, as determined using the HEK-Blue IFN assay in A549 cells (Supplementary Figure S6B).

Crucially, the denaturing PAGE/Urea analysis detected single, well-defined bands for both short viral and Y5 RNAs (Figure 5C). This, together with the results from RNAseq, led us to a hypothesis that dsRNAs arise from the primary product of IVT, coupled with its perfectly complementary antisense strand of equivalent length, produced through promoterless, RNA-dependent transcription. To validate this hypothesis, we compared the migration patterns of IVT short viral and Y5 RNAs with a short viral and Y5 dsRNA control on native PAGE (Figure 5D) and subsequently through native PAGE followed by immuno-northern blot with anti-dsRNA J2 antibody detection (Figure 5E). Both experiments revealed an increased presence of fully dsRNA species in the IVT substrates beginning with 5′-pppA. We also tested the stability of short viral RNAs after transfection in cells by qRT-PCR. The analysis did not reveal significant differences in the abundance of 5′-pppA and 5′-pppG RNAs (Supplementary Figure S7A). Then, to assess the phosphorylation status of their 5′ end, after purification of total RNA from cells transfected either with 5′-pppA or 5′-pppG short viral RNAs, we treated it with XRN1 or RppH combined with XRN1. The XRN1 enzyme degrades dephosphorylated RNAs, while RppH dephosphorylates them. The RNAs were resistant to XRN1 treatment, indicating they still retained 5′-ppp moiety (Supplementary Figure S7A). However, due to higher RppH’s affinity for 5′-pppA RNAs (62), only 5′-pppA RNAs were efficiently cleaved after cotreatment with RppH and XRN1. Finally, we verified the activity and specificity of the used enzymes on IVT RNAs, which were not transfected into cells (Supplementary Figure S7B).

To explore whether the immunogenicity was predominantly derived from dsRNAs, we created short viral RNA by combining the initial 55 nt obtained through IVT with the remaining 21 nt chemically synthesized using splint ligation. Native PAGE analysis demonstrated that both IVT and semisynthetic RNAs were indistinguishable, except for the band attributed to fully dsRNA in the 5′-pppA RNA (Figure 5F). The HEK-Blue IFN assay revealed that the semisynthetic ligated RNA showed no immunogenicity at the concentrations tested (Figure 5G). However, the introduction of minimal quantities of a fully complementary antisense RNA strand (1–5%) triphosphorylated or dephosphorylated, mirroring levels found in IVT RNAs, was sufficient to activate the RIG-I/IFN pathway (Figure 5G). Similar results were obtained for IVT and semisynthetic Y5 RNAs (Supplementary Figure S8A and B).

To further validate the increased presence of dsRNAs in IVT RNAs with 5′-pppA, we performed an RNA pull-down Mass Spectrometry (RP-MS) assay. We identified a significant number of dsRNA-binding proteins, such as ADAR, PKR (EIF2AK2), and DICER1, exclusively in the short viral 5′-pppA RP-MS (Supplementary Figure S9A). Validation with western blot analysis confirmed increased binding of RIG-I, PKR and DHX9 to 5′-pppA RNA (Supplementary Figure S9B). These findings collectively demonstrate that IVT RNA starting with 5′-pppA produces significantly higher levels of immunogenic dsRNA.

### Enhanced dsRNA production initiated by 5′-pppA is largely independent of RNA sequence and structure

**Figure 6.**
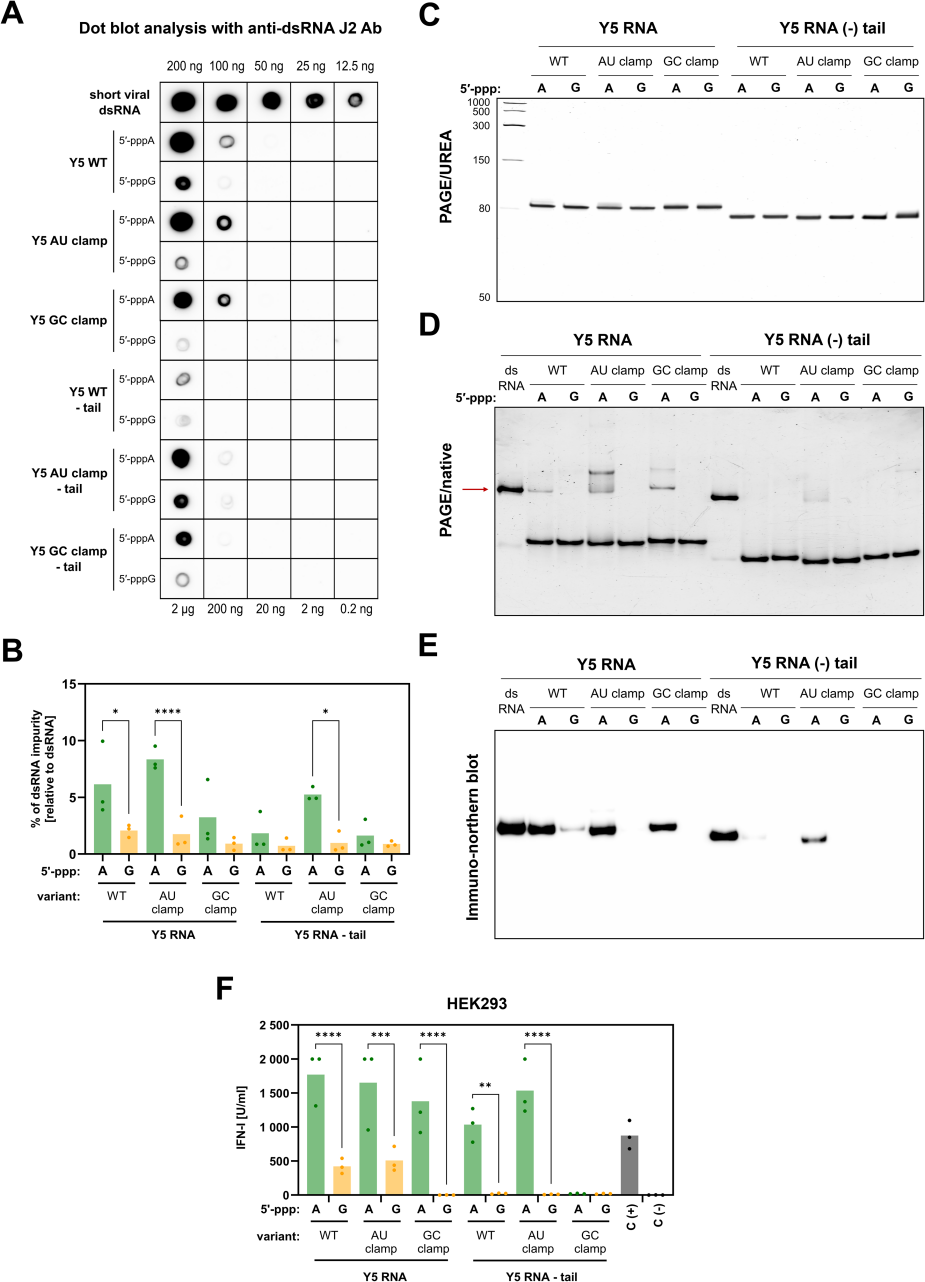
Increased dsRNA production in 5′-pppA is independent of RNA structure and sequence. (**A**) Dot blot analysis of IVT produced variants of Y5 RNA starting from 5′-pppA or 5′-pppG was performed with anti-dsRNA J2 antibody, and (**B**) amount of dsRNA contamination was determined with densitometric analysis (*n* = 3). (**C**) PAGE/Urea analysis of Y5 RNA variants showed single band for each RNA. (**D**) PAGE in native conditions, however, exhibited the presence of additional dsRNA bands. (**E**) Immuno-northern blot using PAGE native gel followed by transfer to the membrane and detection with anti-dsRNA J2 antibody was used to detect the presence of dsRNA formed by annealing with the 78 nt long complementary antisense strand. (**F**) HEK293 cells were transfected with Y5 RNA variants and the concentration of type I IFN in the supernatants was measured using HEK-Blue IFN assay at 24 h *n* = 3). The positive control (C(+)) involved transfection with 100 ng/ml 3p-hpRNA, while the negative control (C(−)) was mock-transfected with lipofectamine alone. (**B**, **F**) Data were compared using two-way ANOVA with Šídák's multiple comparisons test.

To explore why IVT reactions with 5′-pppA result in increased dsRNA production compared to 5′-pppG, we analyzed a series of Y5 RNA mutants (Supplementary Figure S10, Supplementary Table S1). We hypothesized that the sense RNA’s secondary structure and thermodynamic properties might influence dsRNA formation. Mutants were synthesized with either 5′-pppA or 5′-pppG and ended in U-rich tails or no tails, with stem sequences modified to include a six-nucleotide AU or GC clamp (positions from N2:N74 to N7:N69) (Supplementary Figure S10). Dot blot analysis with anti-dsRNA J2 antibody confirmed higher dsRNA levels in RNAs initiated with 5′-pppA than those with 5′-pppG (Figure 5A and B). PAGE/Urea gels showed single RNA bands, consistent with fully sense/antisense source of dsRNA (Figure 6C). Notably, U-rich tail mutants produced more dsRNA than those without (Figure 6A, B, D and E). However, native PAGE and immuno-northern blot (Figure 6D and E) verified increased fully dsRNA species in almost all 5′-pppA mutants. Further assays for RIG-I/IFN pathway activation demonstrated higher IFN production triggered by 5′-pppA RNAs compared to 5′-pppG counterparts (Figure 6F). Notably, Y5 GC clamp RNA lacking a U-rich tail failed to stimulate IFN-I (Figure 6F), implying possible cellular factors limit the immunogenicity of specific 5′-ppp RNAs. These findings indicate that the sense RNA’s thermodynamic properties and structure influence global dsRNA contamination levels in IVT. However, consistent increased dsRNA presence in 5′-pppA RNAs suggests an additional mechanism promoting dsRNA formation specific to 5′-pppA. Additional evidence supporting this molecular mechanism came from IVT reactions of short viral and Y5 RNAs performed in the reaction of the same volumes, where we found more dsRNA contamination in 5′-pppA RNAs, despite the total RNA yield being higher for 5′-pppG RNAs (Supplementary Figure S11A-C).

### Enhanced dsRNA production in long IVT RNAs

**Figure 7.**
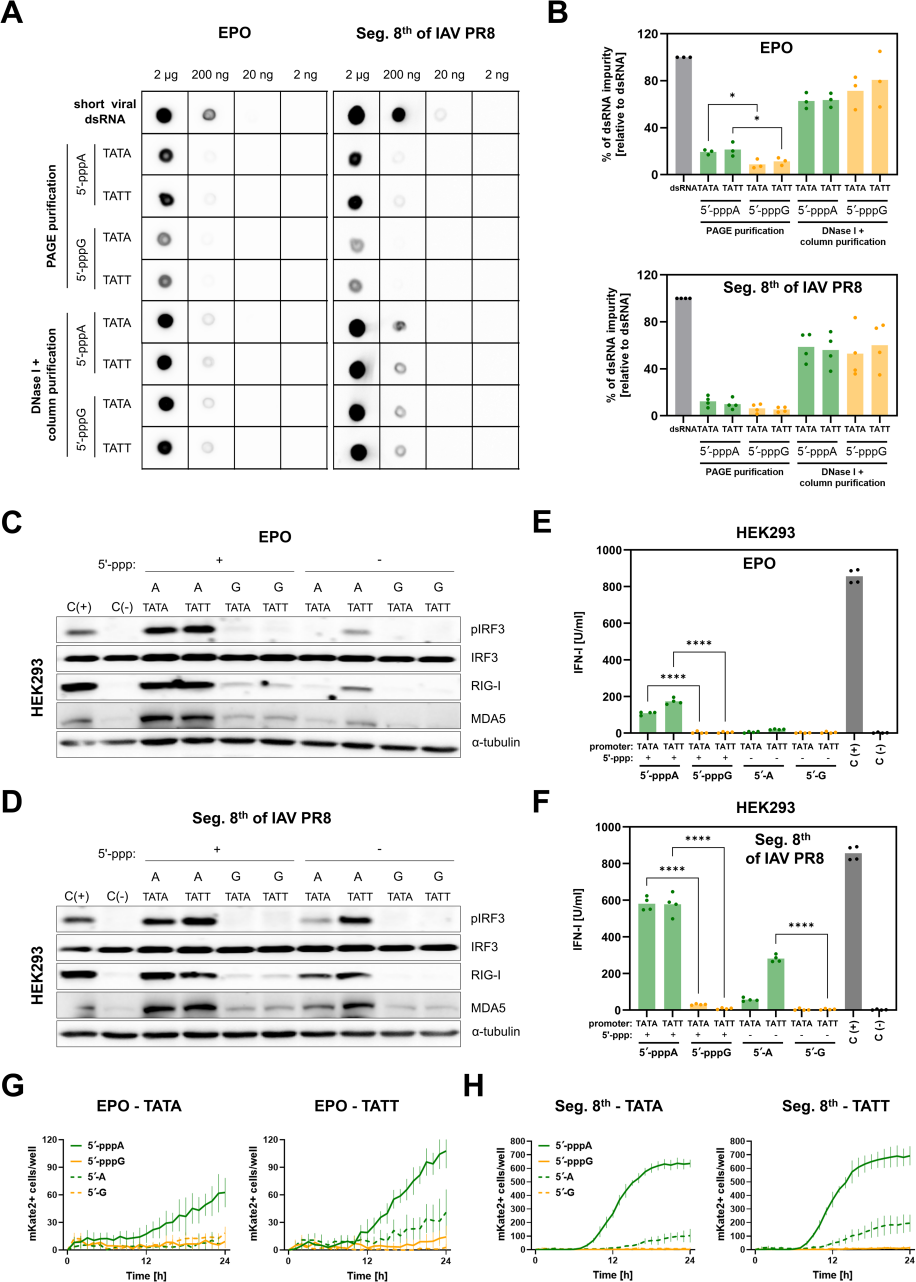
Long IVT-derived 5′-pppA RNAs contain more dsRNA byproducts compared with 5′-pppG RNAs. (**A**) Dot blot analysis of IVT EPO and Seg. 8^th^ of IAV PR8 RNAs starting from 5′-pppA or 5′-pppG and produced either with Class III (TATA) or Class II (TATT) T7 promoter. (**B**) Densitometric analysis (*n* = 3 for EPO RNA, *n* = 4 for Seg. 8^th^ of IAV PR8 RNA) was conducted to assess the level of dsRNA byproducts in IVT-derived RNAs. (**C–F**) RIG-I/IFN pathway responses against EPO and Seg. 8^th^ of IAV PR8 RNAs starting from 5′-pppA or 5′-pppG were assessed with western blot analysis (**C**, **D**) and HEK-Blue IFN assay (**E**, **F**) 24 h after transfection into HEK293 cells (*n* = 4). (**E**, **F**) Data were compared using two-way ANOVA with Šídák's multiple comparisons test. The presence of dsRNA in EPO RNAs (**C**, **E**, **G**) RNAs (**D**, **F**, **H**). dsRNA controls are fully double stranded RNA derived from sense and antisense IVT. The positive control (C(+)) involved transfection with 100 ng/ml 3p-hpRNA, while the negative control (C(−)) was mock-transfected with lipofectamine alone.

Given the widespread use of IVT reaction for generating mRNA for vaccines and future replacement therapies, we aimed to explore whether the terminal nucleotide could influence the production of dsRNAs in longer substrates (Supplementary Table S1). To evaluate this, we synthesized RNAs encoding EPO flanked with UTRs derived from the mRNA sequence of the Moderna SARS-CoV2 vaccine (mRNA-1273) and Segment 8^th^ from the PR8 strain of IAV (Seg. 8^th^ IAV PR8). Elevated levels of dsRNA were observed with anti-dsRNA J2 dot blot for 5′-pppA EPO and Seg. 8^th^ IAV PR8 RNAs purified via PAGE/Urea (Figure 7A and B), although only for EPO mRNA the differences were statistically significant. Interestingly, the type of T7 promoter did not influence this difference. Higher dsRNA presence was noted in both EPO and Seg. 8^th^ IAV PR8 substrates that were only treated with DNase I and purified on a column (Figure 7A and B). This suggests that there might be additional, different length dsRNA by-products generated during IVT. Functional testing in HEK293 and A549 cells demonstrated that both PAGE-purified EPO and Seg. 8^th^ IAV PR8 RNAs triggered a much higher RIG-I/IFN response when starting with 5′-pppA (Figure 7C-H, and Supplementary Figure S12A-D). Moreover, the nature of the T7 promoter in EPO, Seg. 8^th^ IAV PR8 RNAs and short viral RNA did not have a major influence on the immunogenicity of tested RNAs (Figure 7 and Supplementary Figures S12-S13). However, some dephosphorylated 5′-pppA RNAs retained immunogenicity, either due to incomplete dephosphorylation or activation of alternative innate immune signaling pathways.

**Figure 8.**
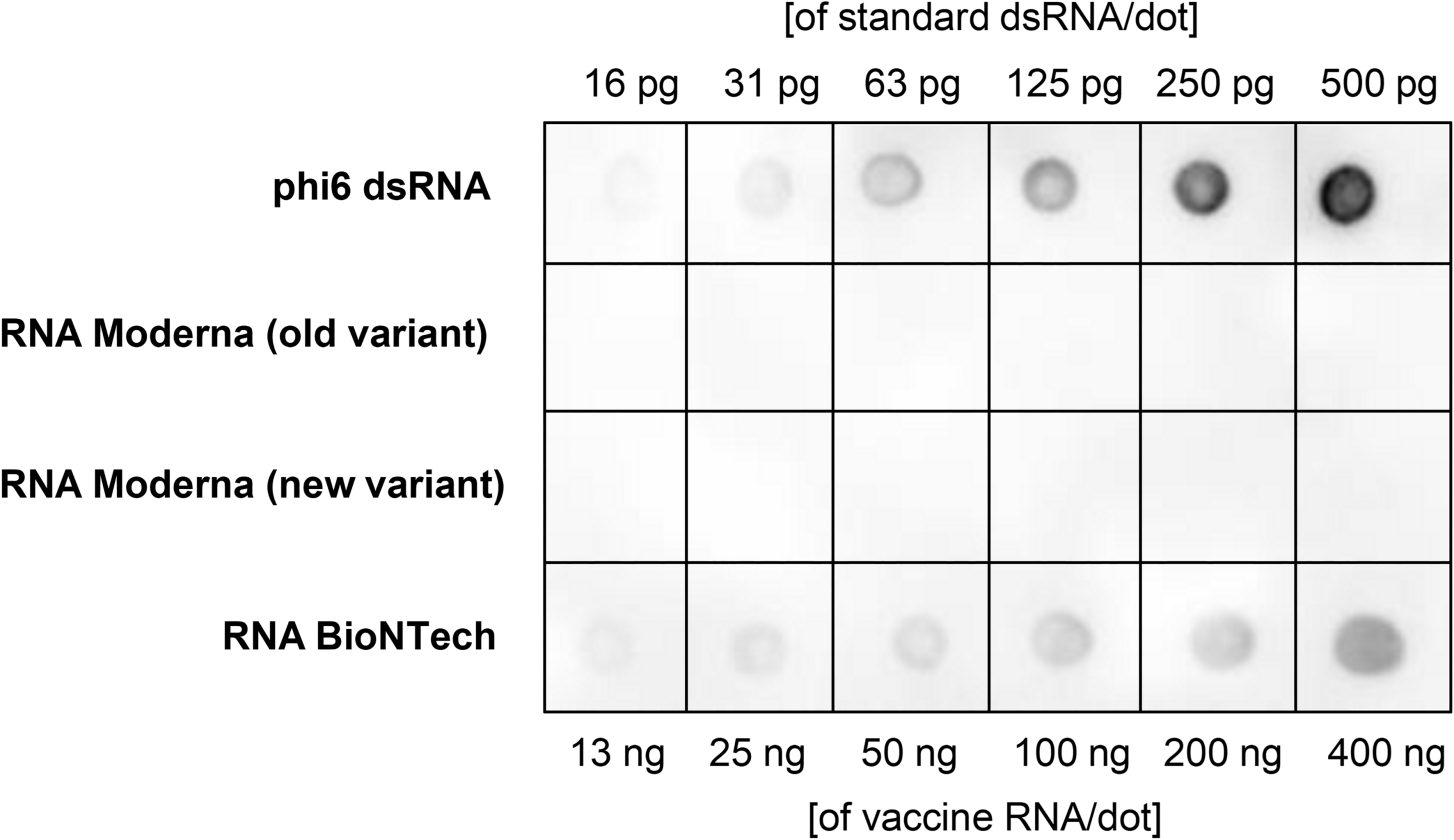
Quantification of dsRNA in mRNAs derived from Moderna and BioNTech SARS-CoV-2 vaccines using the J2 antibody staining. Phi6 dsRNA was used as a positive control standard for J2 antibody detection. The experiment was conducted in triplicate, with a representative image shown. Old variant represents monovalent vaccine, new variant represents bivalent BA.4–5 vaccine.

Finally, we compared dsRNA levels between RNAs extracted from the Moderna and BioNTech SARS-CoV-2 vaccines. Notably, dsRNAs were clearly detectable in the BioNTech RNA vaccine (Figure 8), which utilized co-transcriptional addition of an AG-trinucleotide CleanCap (34). Conversely, the Moderna RNA vaccine, produced by enzymatically incorporating Cap1 on the 5′-pppG-containing IVT RNAs (33), did not exhibit detectable dsRNAs (Figure 8). However, it is important to acknowledge that the disparity in observed dsRNA abundances may be influenced by the utilization of different production and purification protocols by Moderna and BioNTech.

Together, our results reaffirm that IVT generates a considerable amount of dsRNA, and even stringent PAGE purification can lead to increased levels of dsRNA in substrates starting with 5′-pppA.

## Discussion

In our study, we demonstrate the hyperactivation of the RIG-I/IFN type I pathway following transfection with IVT-derived 5′-pppA RNAs in human and mouse cells across a broad range of concentrations. While previous research noted increased RIG-I stimulation by short 5′-pppA dsRNA, this aspect remained unexplored (9). Our findings uncover that the primary driver of this phenomenon is the elevated presence of dsRNA in IVT 5′-pppA RNAs compared to their 5′-pppG counterparts. One study found no difference in the induction of IFN-β when using either fully double-stranded or stem-loop synthetic RNAs containing either 5′-pppA or 5′-pppG (63). However, these RNAs were transfected into cells at a high dose of 1 μg. Thus, it aligns with our findings as fully synthetic RNAs would lack dsRNA by-products, and we observed no distinction in immunogenicity between 5′-pppA or 5′-pppG at the highest concentrations.

For decades, T7 polymerase has been employed to produce substrates for investigating a broad spectrum of RNA biology (18). In recent years, it has enabled production of novel classes of SARS-CoV-2 mRNA vaccines, resulting in saving millions of lives (64). It is worth mentioning that the BioNTech vaccine was administered at 30 μg per dose, while the Moderna vaccine was given at 100 μg per dose (65). Our results indicate a greater presence of dsRNA contaminants in the BioNTech vaccine RNA. This could potentially explain the rationale behind using a lower RNA concentration per dose to mitigate unintended innate immune responses. Thus, as we progress towards integrating IVT-based medications into other areas of medicine (66), a comprehensive understanding of this system and its outputs becomes essential.

Prior research has detected specific dsRNA by-products resulting from the IVT synthesis process that trigger cellular immune responses, which has been demonstrated as a primary activator of the immune pathway (19). These dsRNA by-products are proposed to primarily arise through two distinct mechanisms. Firstly, the RNA transcript synthesized by T7 acts as a template for the RNA-dependent RNA runoff polymerase activity (67). If the 3′-end of the transcript possesses adequate complementarity, it may fold back, leading to the extension of the runoff transcript. This extended RNA can be easily differentiated from the main transcript under denaturing gel electrophoresis. The alternative mechanism involves the formation of dsRNA via promoter-independent transcription from the primary RNA product (19). In shorter RNAs, the size of the antisense molecule closely matches that of the main product, yielding highly immunogenic blunt-end dsRNA with 5′-ppp, which remains indistinguishable through denaturing gel electrophoresis. In longer RNAs, the polymerase can generate shorter dsRNA by-products through promoter-independent transcription. While extensive HPLC-based purification has demonstrated efficacy in separating dsRNA by-products from the main IVT RNA products (28), this approach is not conducive to scaling up due to its high cost. Other strategies to mitigate dsRNA production during IVT include reducing magnesium levels (19) or employing cellulose-based chromatography (29). These approaches do not achieve full elimination of dsRNA. In this study, we illustrate how the selection of the 5′ nucleoside during IVT significantly influences immunogenicity levels by affecting dsRNA generation. Specifically, RNA with a 5′-pppA motif yields elevated quantities of dsRNA and provides abundant perfect RIG-I agonists. We have also shown that both the stability of the sense transcript and the presence of a 3′ overhang affect dsRNA formation, which aligns with previous findings indicating that unintended transcription primarily occurs when the correct product cannot form stable secondary structures at the 3′-end (24). However, in most cases, we consistently observed higher levels of dsRNA contamination in cognate RNAs beginning with 5′-pppA compared to those starting with 5′-pppG. Although the precise molecular mechanism remains unknown, we hypothesize that this could be due to inefficient engagement of T7 polymerase with templates designed to produce 5′-pppA RNA compared to 5′-pppG transcripts. This could allow the T7 to reengage with the 3′ end of the sense RNA and initiate promoterless, RNA-dependent transcription.

Furthermore, we have developed a novel, highly sensitive assay to investigate the immunogenicity of RNAs both *ex vivo* and *in vivo*. Our mouse model, in which the IFN-β gene was substituted with the mKate2 reporter, proved effective in detecting immunogenic RNAs in BMDMs as well as in the cells derived from liver and spleen. While a previous study had utilized a mouse expressing luciferase fused with IFN-β (68), our model allows for the analysis of primary effects of PAMPs, as the most potent type 1 IFN is not produced (69), thus avoiding triggering downstream JAK/STAT pathway. At the same time, alpha IFNs, despite exhibiting 20–30 times lower affinity to IFN type 1 receptors than IFN-β (69), are still expressed, enabling the mice's normal development and physiological function.

IVT reaction is a standard method for producing RNA-based vaccines and therapeutics. These RNAs are typically capped and polyadenylated to ensure proper functionality. However, incomplete capping may result in RNAs with a triphosphate group at the 5′ end, which can trigger an innate immune response (70). Our results demonstrate that IVT RNAs with 5′-pppA, in contrast to 5′-pppG, induce a more robust type I IFN response in *in vitro*, *ex vivo* and *in vivo* settings (Figures 2–7). The primary limitations of the study are the use of only unmodified RNAs, the reliance on a single T7 RNA polymerase, and the testing of a selection of transcripts. Further research is necessary to determine whether this phenomenon applies to capped, polyadenylated, and N1-methylpseudouridine-incorporated mRNAs, and, if so, how it impacts capping, translation, and the effectiveness of the adaptive immune response. Lastly, the differences we identified between 5′-pppA and 5′-pppG RNAs should be considered when designing RIG-I agonists, which can be used to trigger broad antiviral responses (Supplementary Figure S3) (71,72) or promote IFN-I-dependent apoptosis, potentially serving as anti-cancer agents (73).

In summary, our study reveals a significant disparity in immunogenicity between 5′-pppA-containing IVT RNAs and their 5′-pppG counterparts. We have observed that IVT RNAs with 5′-pppA exhibit heightened immunogenicity, characterized by increased levels of dsRNAs and activation of the RIG-I signaling pathway. These findings have important implications for both research and medical applications, shedding light on the mechanisms underlying IVT RNA immunogenicity and offering potential avenues for optimizing therapeutic interventions.

## Supplementary Material

gkae1252_Supplemental_File

## Data Availability

The RNAseq data presented in the study have been deposited in the Sequence Read Archive repository (BioProject accession number #PRJNA1054022). The mass spectrometry proteomics data have been deposited to the ProteomeXchange Consortium via the PRIDE (74) partner repository with the dataset identifier PXD057761.
