# Supplementary material for: 5′ terminal nucleotide determines the immunogenicity of IVT RNAs
Source: Nucleic Acids Res. 2024 Dec 19;53(3):gkae1252. doi: 10.1093/nar/gkae1252 (PMC11797061; doi:10.1093/nar/gkae1252)
Supplement: gkae1252_Supplemental_File [file gkae1252_supplemental_file.docx]

# Supplementary Data

# 5′ terminal nucleotide determines the immunogenicity of IVT RNAs

Magdalena Wolczyk^1*^, Jacek Szymanski^1*^, Ivan Trus^1*^, Zara Naz^1^, Tola Tame^1^, Agnieszka Bolembach^1^, Nila Roy Choudhury^1,2^, Karolina Kasztelan^1^, Juri Rappsilber^3^, Andrzej Dziembowski^1^, Gracjan Michlewski^1^

* These authors contributed equally to this work.

^1^ International Institute of Molecular and Cell Biology in Warsaw, Poland

^2^ MRC Human Genetics Unit, Institute of Genetics and Cancer, University of Edinburgh, Western General Hospital, Edinburgh, UK

^3^ Department of Biotechnology, Technische Universität Berlin, Berlin, Germany

Correspondence should be addressed to G.M. (gmichlewski@iimcb.gov.pl)

**
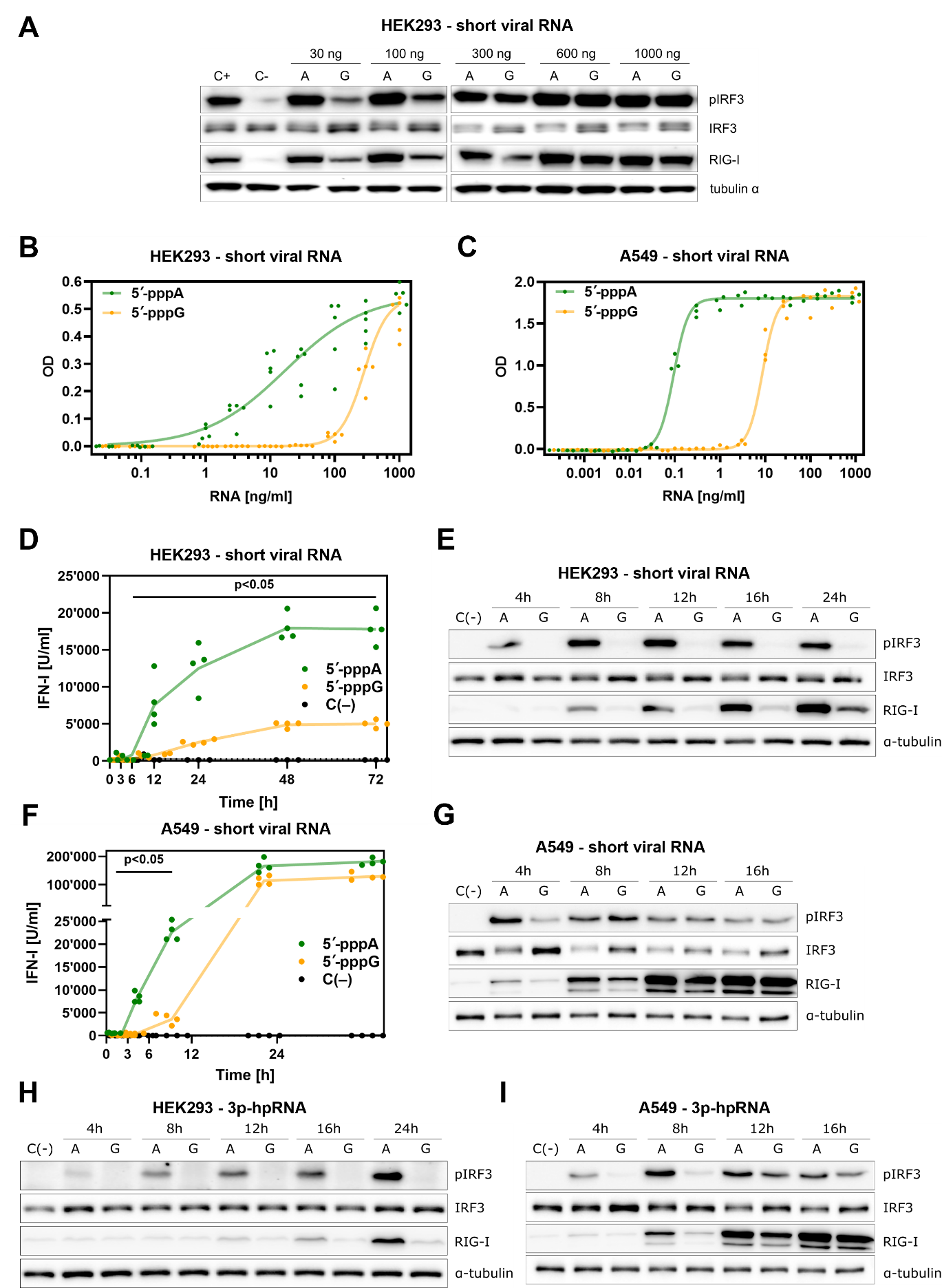
**

**Supplementary figure S1. IVT RNAs starting with 5′-pppA are more immunogenic than those starting with 5′-pppG.** (A) Analysis of IRF3 phosphorylation and RIG-I expression assessed by western blot analysis in HEK293 cells after transfection with different concentrations of short viral 5′-pppA *vs.* 5′-pppG RNAs ranging from 30 ng/ml to 1000 ng/ml. The cells were incubated for 24 h, then cell lysis was performed. (B, C) The apparent dissociation constant (K_d_) was estimated for each RNA variant (n = 5). For virus-derived short RNA introduced into A549 cells, K_d_ was estimated to equal 0.094 ng/ml (3.85 pM) for 5′-pppA RNA (95% confidence limits, 0.089–0.099 ng/ml; R^2^_adj_ = 0.9973). For 5′-pppG RNA K_d_ was estimated to be 8.677 ng/ml (355.8 pM) (95% confidence limits, 8.377–8.975 ng/ml; R^2^_adj_ = 0.9987). In the case of HEK293 cells, the estimated K_d_ values were 17.0 ng/ml for 5′-pppA (95% confidence limits, 9.6–41.8 ng/ml; R^2^_adj_ = 0.9175) and 282.8 ng/ml for 5′-pppG RNA variants (95% confidence limits, 280.9–284.8 ng/ml; R^2^_adj_ = 0.9999). (D, F) Kinetics of type I IFN production in HEK293 and A549 cells treated with RNA representing fragment of the IAV genome at a concentration of 100 ng/ml (n = 4). The dotted line represents lower quantification limit. The positive control (C(+)) involved transfection with 100 ng/ml 3p-hpRNA, while the negative control (C(−)) was mock-transfected with lipofectamine alone. Upon log-transformation data were compared using two-way ANOVA with Šídák’s multiple comparisons test. (E, G) Kinetics of IRF3 phosphorylation and RIG-I expression assessed by Western blot analysis in HEK293 and A549 cells treated with 5′-pppA *vs.* 5′-pppG short viral RNA at a concentration of 100 ng/ml. (H, I) Kinetics of IRF3 phosphorylation and RIG-I expression assessed by Western blot analysis in HEK293 and A549 cells treated with 5′-pppA *vs.* 5′-pppG 3p-hpRNA at a concentration of 100 ng/ml.


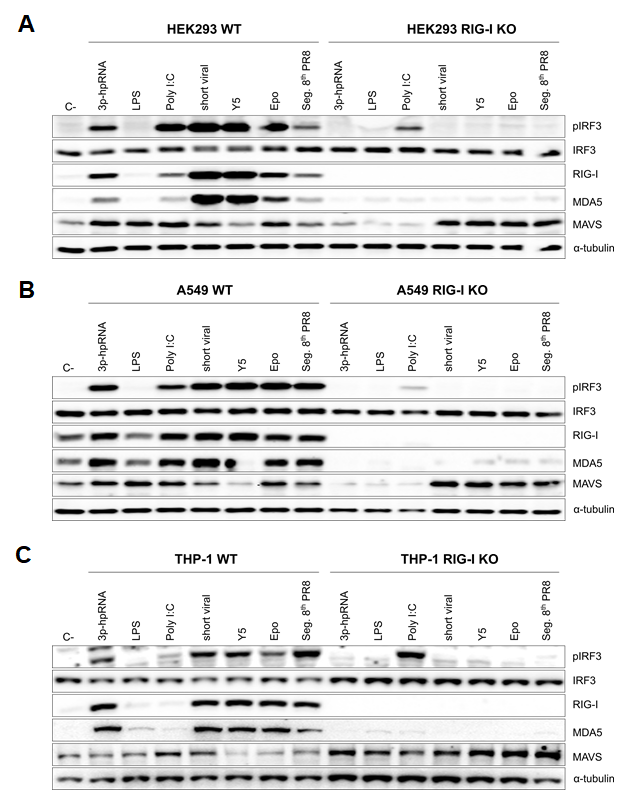


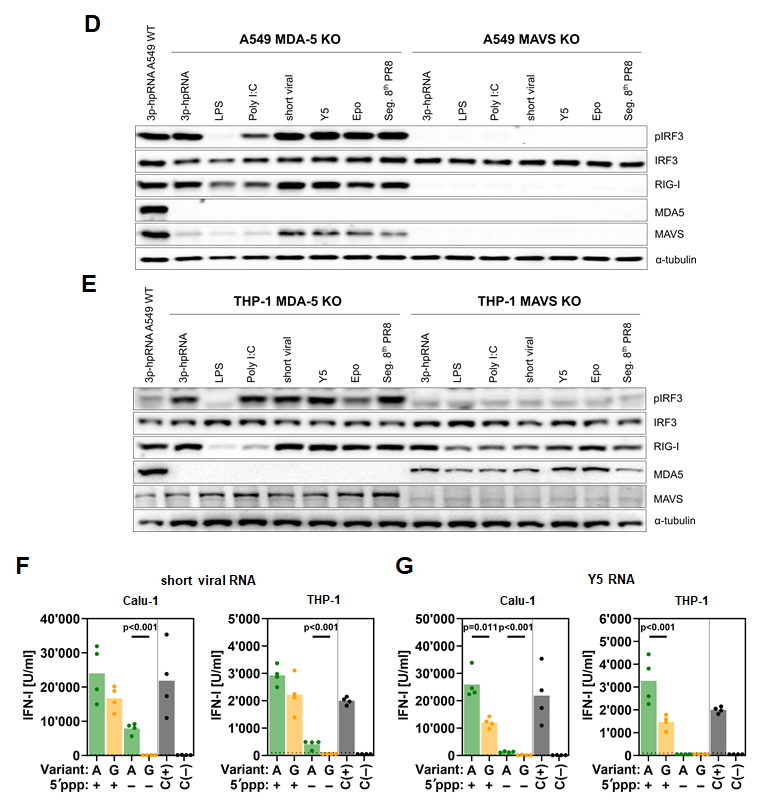


**Supplementary Figure S2.** **RNA used in the study mostly act through RIG-I signalling pathway.** (A-E) RIG-I/IFN pathway responses against RNAs starting from 5′-pppA and 5′-pppG in RIG-I KO cells (A-C) and in MDA-5 or MAVS KO cells (D, E). (F, G) Comparison of IFN responses against RNAs starting from 5′-pppA and 5′-pppG in cell cultures of human origin (THP-1 and Calu1). RNAs representing a fragment of the IAV genome (short viral RNA) and Pol III Y5 transcript were transfected into cells at a concentration of 100 ng/ml (n = 4). The positive control (C(+)) involved transfection with 100 ng/ml 3p-hpRNA, while the negative control (C(−)) was mock-transfected with lipofectamine alone. (A-E) The IRF3 phosphorylation pattern/RIG-I expression and (F, G) the concentrations of type I IFN in the supernatants were assessed using Western blot analysis and HEK-Blue assay, respectively, after 24 hours of incubation. (F, G) Upon log-transformation data were compared using two-way ANOVA with Šídák’s multiple comparisons test.


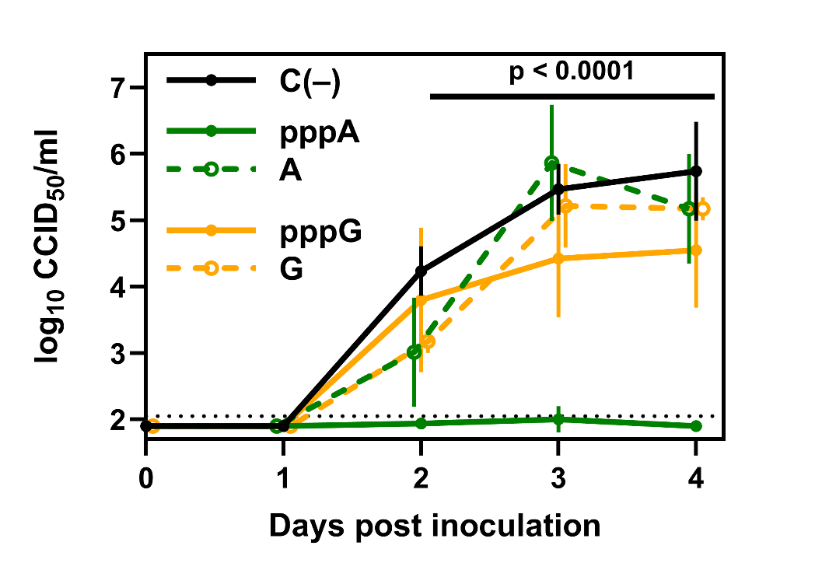


**Supplementary Figure S3.** **5′-pppA RNA is capable of lowering virus replication *in vitro* compared to 5′-pppG variant and dephosphorylated RNAs.** Short viral RNA with either 5′-pppA and 5′-pppG or their dephosphorylated versions were transfected 24 h prior infection with IAV A/PR/8/34_NS1(R38A/K41A) at an MOI of 0.0001. Whiskers represent the standard deviation (SD) from four biological replicates. A significant reduction in virus replication was observed for 5′-pppA RNA with P *<* 0.0001 in two-way ANOVA followed by Dunnett’s multiple comparison test to mock-infected cells (C(−)).


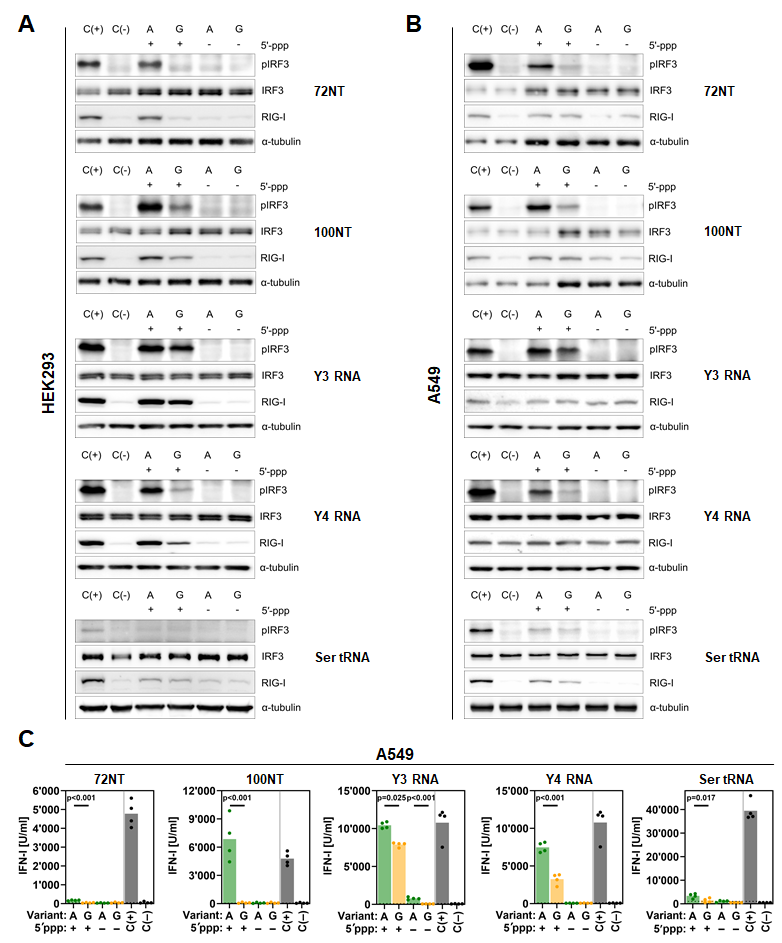


**Supplementary Figure S4. Various IVT RNAs starting with 5′-pppA are more immunogenic than those starting with 5′-pppG.** (A, B) RIG-I/IFN responses against different RNAs (sequences in Supplementary Table S1) starting from 5′-pppA and 5′-pppG were assessed with Western blot analysis 24 hours after transfection in HEK293 cells (A) and 8 hours in A549 cells (B). (C) The concentrations of type I IFN in the supernatants 8 hours after transfection in A549 cells were assessed with HEK-Blue assay (n = 4). All RNAs were transfected into cells at a concentration of 100 ng/ml. The positive control (C(+)) involved transfection with 100 ng/ml 3p-hpRNA, while the negative control (C(−)) was mock-transfected with lipofectamine alone.

**
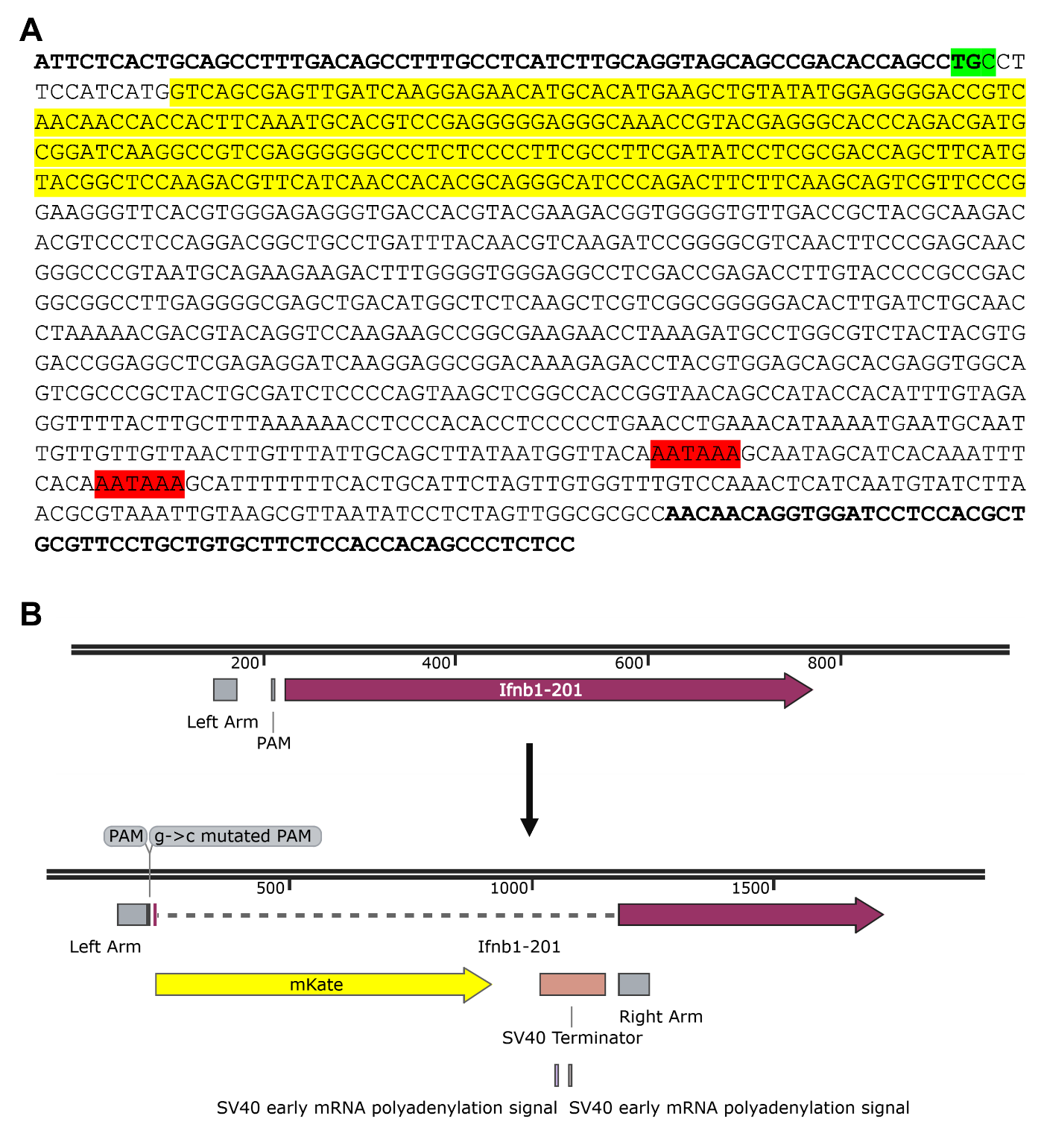
**

**Supplementary Figure S5.** **Schematics of generating mice line harboring a fluorescent marker mKate2 in the IFN-β gene.** (A) The nucleotide sequence of optimized mKate2 marker (highlighted in yellow), mutated protospacer-adjacent motif (highlighted in green), SV40 early mRNA polyadenylation signal (highlighted in red), and flanking regions of the murine genome (highlighted in bold). (B) The genomic context corresponds to *Mus musculus* strain C57BL/6J (the GRCm39 reference genome, chromosome #4, positions NC_000070.7:c88441083-88440949).

**
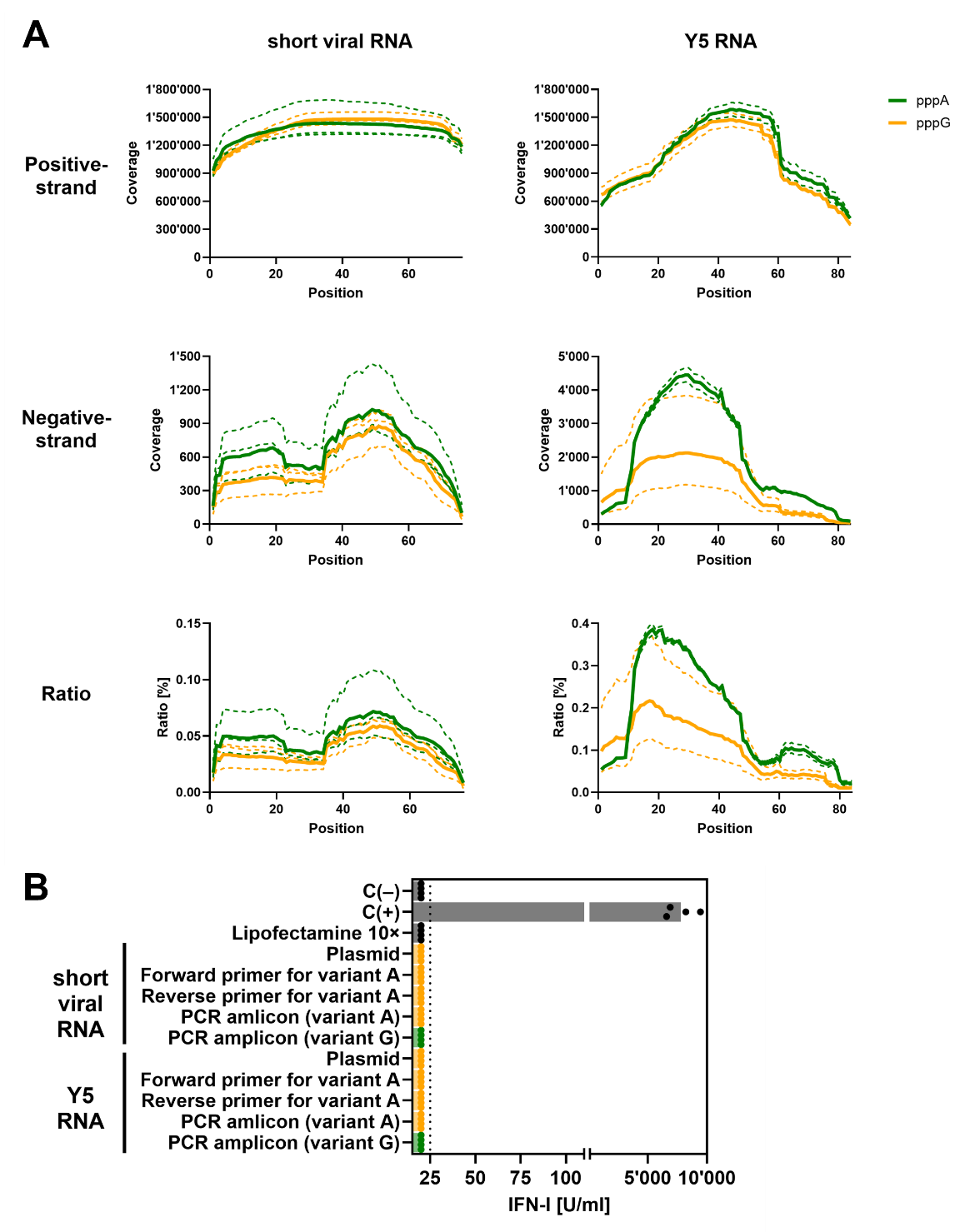
**

**Supplementary Figure S6. Full-length antisense strand of IVT RNAs is detected by NGS. Results of stranded quantification of RNA.** (A) Coverage depth for NGS data was separately calculated for reads mapped either to positive or negative, shown as individual dashed lines. Geometric mean values are represented by solid lines. The ratio of negative to positive strand reads was estimated for each replicate (n = 3 for short viral RNA; n = 2 for Y5 RNA). (B) Reagents used for transfection were tested in A549 cells with the HEK-Blue assay for IFN-I induction. Primers, original plasmids, and PCR amplicons were tested at a concentration of 100 ng/ml (n = 4). The positive control (C(+)) involved transfection with 100 ng/ml 3p-hpRNA, while the negative control (C(−)) was mock-transfected with lipofectamine alone. Lipofectamine 2000 was used at transfections at 1× concentration (2 µl/ml), no IFN-I induction was observed at higher (10×) concentrations.

**
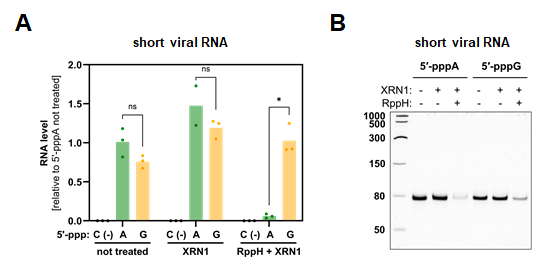
**

**Supplementary Figure S7. 5′-pppA and 5′-pppG short viral RNAs exhibit similar stability and remain triphosphorylated after transfection into HEK293 cells as shown by *ex vivo* (A) and *in vitro* (B) analysis of RNA 5′-end phosphorylation state.** (A) HEK293 cells were transfected with tested RNAs, then total RNA isolation followed by XRN1 or RppH combined with XRN1 treatment was performed. RT-qPCR was then used to assess the level of short viral RNA. Mean values of three biological replicates are shown. Data were compared using two-way ANOVA with Šídák’s multiple comparisons test. (B) IVT derived short viral RNAs were treated with XRN1 or RppH and XRN1 and then PAGE/Urea was performed.


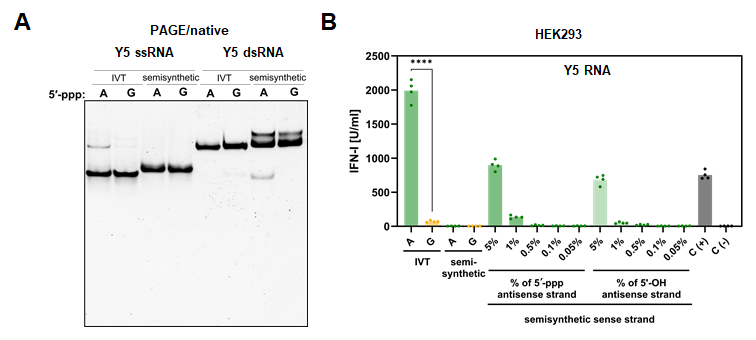


**Supplementary Figure S8. Splint-ligated semisynthetic Y5 RNAs exhibit immunogenic potential only after annealing with 78 nt long complementary antisense RNA strand.** (A) PAGE in native conditions of IVT Y5 RNAs and semisynthetic Y5 RNAs produced by splint ligation revealed dsRNA presence in IVT 5′-pppA, but not in semisynthetic counterparts. (B) To assess the immunogenic potential of splint-ligated RNAs, an IVT-produced antisense RNA, with or without triphosphate moieties, was added to the sense splint-ligated RNA. The positive control (C(+)) involved transfection with 100 ng/ml 3p-hpRNA, while the negative control (C(−)) was mock-transfected with lipofectamine alone. The concentration of type I IFN in the supernatants was measured using HEK-Blue IFN assay at 24 hours after transfection (n = 4).

**
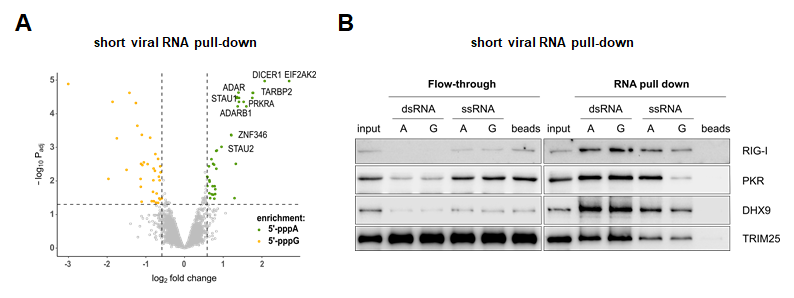
**

**Supplementary Figure S9. dsRNA binding proteins were found to be enriched with 5′-pppA IVT RNA.** (A) To assess interactome differences of 5′-pppA and 5′-pppG IVT short viral RNAs, RNA pull-down assay analysed with LC-MS/MS was performed. The volcano plot (A) illustrates protein enrichment based on the 5′ terminal nucleotide. Proteins enriched with 5′-pppA are highlighted in green, while those enriched with 5′-pppG are shown in orange. Double-stranded RNA-binding proteins are indicated on the plot. (B) RNA pull-down assay followed by Western blot analysis was conducted to validate the binding of PKR (EIF2AK2) – double-stranded RNA-binding protein identified through mass spectrometry (MS) analysis.


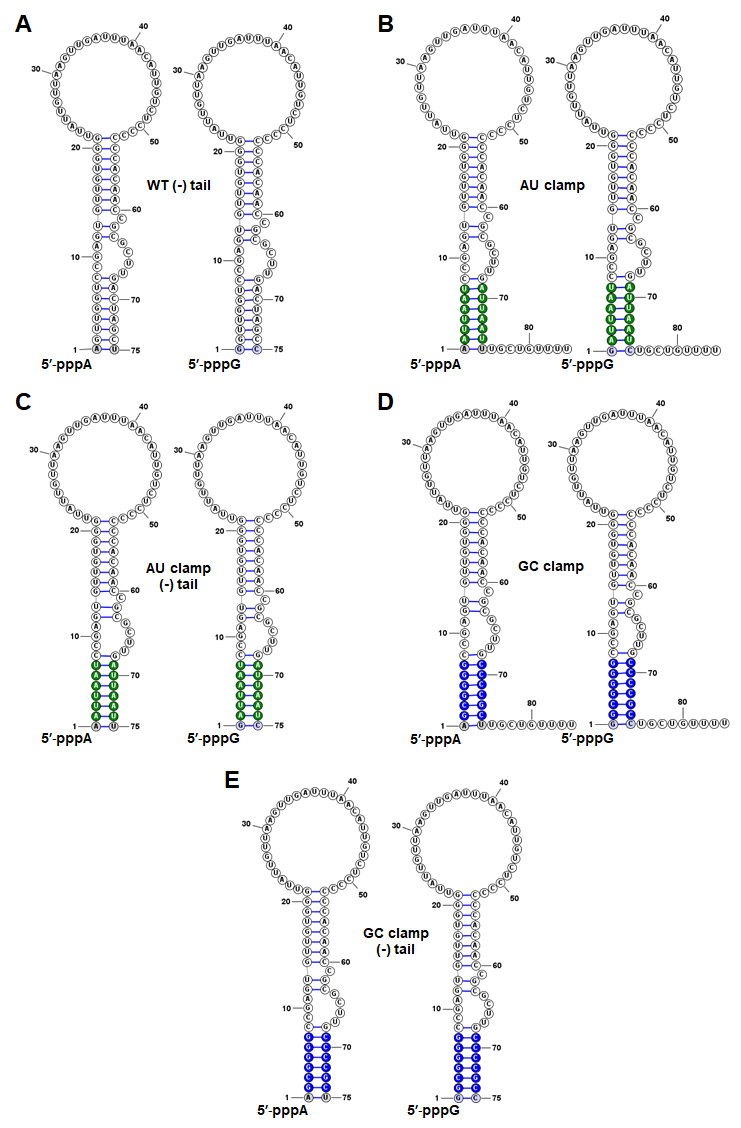


**Supplementary Figure S10. Schemetic representation of Y5 RNA structural and sequence mutants.** Secondary structures of Y5 RNA mutants were predicted using the MFE algorithm in RNAstructure 6.4 software. Mutants starting with either 5′-pppA or 5′-pppG: (A) Y5 wild type RNA without a U-rich tail; (B) Y5 RNA with introduction of a six-nucleotide AU clamp (highlighted in green) with a U-rich tail; (C) Y5 RNA with a six-nucleotide AU clamp (highlighted in green) without a U-rich tail; (D) Y5 RNA with introduction of a six-nucleotide GC clamp (highlighted in blue) with U-rich tail; and (E) Y5 RNA with a six-nucleotide GC clamp (highlighted in blue) and without a U-rich tail.


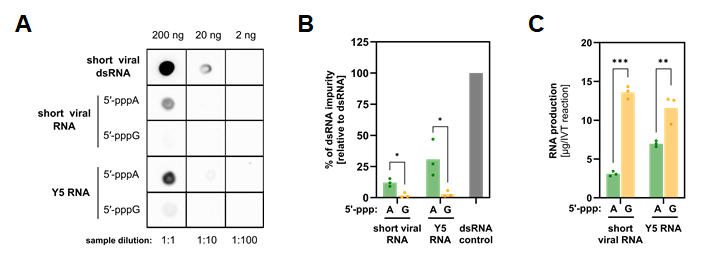


**Supplementary Figure S11. *In vitro* transcription of 5′-pppA RNA results in lower production yield and higher dsRNA contamination.** (A) Dot-blot analysis of IVT short viral and Y5 RNA starting from 5′-pppA or 5′-pppG was performed with anti-dsRNA J2 antibody. Equal volumes of purified RNA were applied to the membrane directly after IVT production, without normalization for RNA concentration. (B) Densitometric analysis was performed to assess the level of dsRNA contamination in purified RNAs after IVT (n = 3). (C) Yield of short viral and Y5 RNA production initiated with 5′-pppA or 5′-pppG, based on A260 RNA concentration measurements (n = 3). Data were compared using two-way ANOVA with Šídák’s multiple comparisons test.


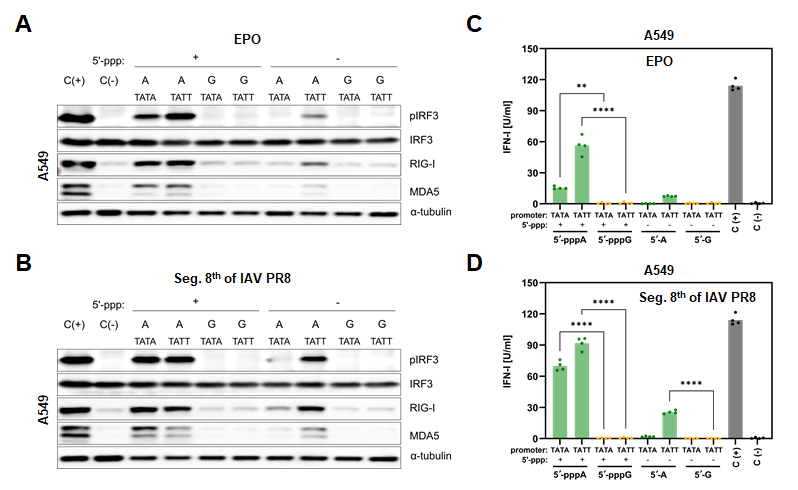


**Supplementary Figure S12. Long IVT 5′-pppA RNAs are more immunogenic then 5′-pppG RNAs after transfection into A549 cells.** RIG-I/IFN pathway responses against EPO and Seg. 8^th^ of IAV PR8 RNAs starting from 5′-pppA or 5′-pppG were assessed with Western blot analysis (A, B) and HEK Blue IFN assay (C, D) 8 h after transfection into A549 cells (n = 4). The positive control (C(+)) involved transfection with 100 ng/ml 3p-hpRNA, while the negative control (C(−)) was mock-transfected with lipofectamine alone. (C, D) Data were compared using two-way ANOVA with Šídák’s multiple comparisons test.


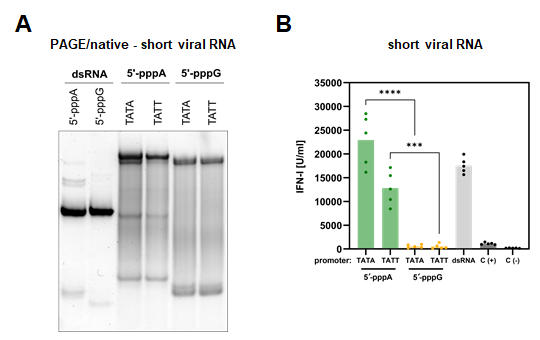


**Supplementary Figure S13. Increased production of dsRNA in 5′-pppA IVT RNA is independent of T7 promoter type.** (A) Native PAGE analysis of IVT control dsRNAs (produced by annealing of sense and antisense RNA strands) and IVT short viral RNAs starting from either 5′-pppA or 5′-pppG, generated using either Class III (TATA) or Class II (TATT) T7 promoters. (B) The immunogenicity of IVT RNAs was evaluated by measuring type I IFN levels in supernatants from HEK293 cells 24 hours post-transfection using the HEK Blue IFN assay (n = 5). The positive control (C(+)) involved transfection with 100 ng/ml 3p-hpRNA, while the negative control (C(−)) was mock-transfected with lipofectamine alone. Data were compared using two-way ANOVA with Šídák’s multiple comparisons test.

**Supplementary Table S1.** RNA transcripts assessed in the current study.

| Transcript | RNA variant | Sequence, 5′-3′ |
| --- | --- | --- |
| short viral | 5′-pppA | AGCAAAAGCAGGGUGACAAAGACAUAAUGGAUCCAAACACUGUGUCAAGCUUUCAGGUAGAUUGCUUUCUUUGGCU |
|  | 5′-pppG | GGCAAAAGCAGGGUGACAAAGACAUAAUGGAUCCAAACACUGUGUCAAGCUUUCAGGUAGAUUGCUUUCUUUGGCC |
| short viral antisense | antisense to 5′-pppA | AGCCAAAGAAAGCAAUCUACCUGAAAGCUUGACACAGUGUUUGGAUCCAUUAUGUCUUUGUCACCCUGCUUUUGCU |
|  | antisense to 5′-pppG | GGCCAAAGAAAGCAAUCUACCUGAAAGCUUGACACAGUGUUUGGAUCCAUUAUGUCUUUGUCACCCUGCUUUUGCC |
| Y5 WT | 5′-pppA | AGUUGGUCCGAGUGUUGUGGGUUAUUGUUAAGUUGAUUUAACAUUGUCUCCCCCCACAACCGCGCUUGACUAGCUUGCUGUUUU |
|  | 5′-pppG | GGUUGGUCCGAGUGUUGUGGGUUAUUGUUAAGUUGAUUUAACAUUGUCUCCCCCCACAACCGCGCUUGACUAGCCUGCUGUUUU |
| 78 nt Y5 antisense | antisense to 5′-pppA | GCAAGCUAGUCAAGCGCGGUUGUGGGGGGAGACAAUGUUAAAUCAACUUAACAAUAACCCACAACACUCGGACCAACU |
|  | antisense to 5′-pppG | GCAGGCUAGUCAAGCGCGGUUGUGGGGGGAGACAAUGUUAAAUCAACUUAACAAUAACCCACAACACUCGGACCAACC |
| Y5 AU clamp | 5′-pppA | AAUUAAUCCGAGUGUUGUGGGUUAUUGUUAAGUUGAUUUAACAUUGUCUCCCCCCACAACCGCGCUUGAUUAAUUUGCUGUUUU |
|  | 5′-pppG | GAUUAAUCCGAGUGUUGUGGGUUAUUGUUAAGUUGAUUUAACAUUGUCUCCCCCCACAACCGCGCUUGAUUAAUCUGCUGUUUU |
| Y5 GC clamp | 5′-pppA | AGCGGGGCCGAGUGUUGUGGGUUAUUGUUAAGUUGAUUUAACAUUGUCUCCCCCCACAACCGCGCUUGCCCCGCUUGCUGUUUU |
|  | 5′-pppG | GGCGGGGCCGAGUGUUGUGGGUUAUUGUUAAGUUGAUUUAACAUUGUCUCCCCCCACAACCGCGCUUGCCCCGCCUGCUGUUUU |
| Y5 WT (-) tail | 5′-pppA | AGUUGGUCCGAGUGUUGUGGGUUAUUGUUAAGUUGAUUUAACAUUGUCUCCCCCCACAACCGCGCUUGACUAGCU |
|  | 5′-pppG | GGUUGGUCCGAGUGUUGUGGGUUAUUGUUAAGUUGAUUUAACAUUGUCUCCCCCCACAACCGCGCUUGACUAGCC |
| Y5 WT (-) tail antisense | antisense to 5′-pppA | AGCUAGUCAAGCGCGGUUGUGGGGGGAGACAAUGUUAAAUCAACUUAACAAUAACCCACAACACUCGGACCAACU |
| Y5 AU clamp (-) tail | 5′-pppA | AAUUAAUCCGAGUGUUGUGGGUUAUUGUUAAGUUGAUUUAACAUUGUCUCCCCCCACAACCGCGCUUGAUUAAUU |
|  | 5′-pppG | GAUUAAUCCGAGUGUUGUGGGUUAUUGUUAAGUUGAUUUAACAUUGUCUCCCCCCACAACCGCGCUUGAUUAAUC |
| Y5 GC clamp (-) tail | 5′-pppA | AGCGGGGCCGAGUGUUGUGGGUUAUUGUUAAGUUGAUUUAACAUUGUCUCCCCCCACAACCGCGCUUGCCCCGCU |
|  | 5′-pppG | GGCGGGGCCGAGUGUUGUGGGUUAUUGUUAAGUUGAUUUAACAUUGUCUCCCCCCACAACCGCGCUUGCCCCGCC |
| 72NT | 5′-pppA | AGUAGAAACAAGGGUGUUUUUUAGUAGACACAGUGUUUGGAUCCAUUAUGUUUUUGUCACCCUGCUUUUGCU |
|  | 5′-pppG | GGUAGAAACAAGGGUGUUUUUUAGUAGACACAGUGUUUGGAUCCAUUAUGUUUUUGUCACCCUGCUUUUGCC |
| 100NT | 5′-pppA | AGUAGAAACAAGGGUGUUUUUUAGUACUAAAUAAGCUGAAACGAGAAAGUGCUUGACACAGUGUUUGGAUCCAUUAUGUUUUUGUCACCCUGCUUUUGCU |
|  | 5′-pppG | GGUAGAAACAAGGGUGUUUUUUAGUACUAAAUAAGCUGAAACGAGAAAGUGCUUGACACAGUGUUUGGAUCCAUUAUGUUUUUGUCACCCUGCUUUUGCC |
| Y3 | 5′-pppA | GGCUGGUCCGAGUGCAGUGGUGUUUACAACUAAUUGAUCACAACCAGUUACAGAUUUCUUUGUUCCUUCUCCACUCCCACUGCUUCACUUGACUAGCCUUUU |
|  | 5′-pppG | GGCUGGUCCGAGUGCAGUGGUGUUUACAACUAAUUGAUCACAACCAGUUACAGAUUUCUUUGUUCCUUCUCCACUCCCACUGCUUCACUUGACUAGCCUUUU |
| Y4 | 5′-pppA | GGCUGGUCCGAUGGUAGUGGGUUAUCAGAACUUAUUAACAUUAGUGUCACUAAAGUUGGUAUACAACCCCCCACUGCUAAAUUUGACUGGCUUUUU |
|  | 5′-pppG | GGCUGGUCCGAUGGUAGUGGGUUAUCAGAACUUAUUAACAUUAGUGUCACUAAAGUUGGUAUACAACCCCCCACUGCUAAAUUUGACUGGCUUUUU |
| tRNA | 5′-pppA | AGAGAGGCCUGGCCGAGUGGUUAAGGCGAUGGACUGCUAAUCCAUUGUGCUCUGCACGCGUGGGUUCGAAUCCCAUCCUCGUUG |
|  | 5′-pppG | GGAGAGGCCUGGCCGAGUGGUUAAGGCGAUGGACUGCUAAUCCAUUGUGCUCUGCACGCGUGGGUUCGAAUCCCAUCCUCGUCG |
| Seg. 8^th^ of  IAV | 5′-pppA | AGUAGAAACAAGGGUGUUUUUUAGUACUAAAUAAGCUGAAACGAGAAAGUUCUUAUCUCUUGCUCCACUUCAAGCAAUAGAUGUAAGGCUUGCAUAAAUGUUAUUUGCUCAAAACUAUUCUCUGUUAUCUUCAGUUUGUGUCUCACUUCUUCAAUCAACCAUCUUAUUUCUUCAAACUUCUGACCUAAUUGUUCCCGCCAUUUCUCGUUUCUGUUUUGGAGUGAGUGGAGGUCUCCCAUUCUCAUUACUGCUUCUCCAAGCGAAUCUCUGUAGAGUUUCAGAGACUCGAACUGUGUUAUCAUUCCAUUCAAGUCCUCCGAUGAGGACUCCAACUGCAUUUUUGACAUCCUCAGCAGUAUGUCCUGGAAGAGAAGGCAAUGGUGAAAUUUCGCCAACAAUUGCUCCCUCUUCGGUGAAAGCCCUUAGCAAUAUUAGAGUCUCCAGCCGGUCAAAAAUCACACUGAAGUUCGCUUUCAGUAUGAUGUUCUUAUCCAUGAUCGCCUGGUCCAUUCUGAUACAAAGAGGGCCUGCCACUUUCUGCUUGGGUAUGAGCAUGGACCAGUCCCUUGACAUUUCCUCAAGAGUCAUGUCAGUUAGGUAACGCGACGCAGGUACAGAGGCCAUGGUCAUUUUAAGUGCCUCAUCGGAUUCUUCUUUCAGAAUCCGCUCCACUAUCUGCUUUCCAGCACGUGUGGCUGUCUUGAUGUCCAGACCGAGAGUACUGCCCCUUCCUCUUAGGGAUUUCUGAUCUCGGCGAAGCCGAUCAAGGAAUGGGGCAUCACCUAGUUCUUGGUCUGCAACUCGUUUGCGGACAUGCCAAAGAAAGCAAUCUACCUGAAAGCUUGACACAGUGUUUGGAUCCAUUAUGUUUUUGUCACCCUGCUUUUGCU |
|  | 5′-pppG | GGUAGAAACAAGGGUGUUUUUUAGUACUAAAUAAGCUGAAACGAGAAAGUUCUUAUCUCUUGCUCCACUUCAAGCAAUAGAUGUAAGGCUUGCAUAAAUGUUAUUUGCUCAAAACUAUUCUCUGUUAUCUUCAGUUUGUGUCUCACUUCUUCAAUCAACCAUCUUAUUUCUUCAAACUUCUGACCUAAUUGUUCCCGCCAUUUCUCGUUUCUGUUUUGGAGUGAGUGGAGGUCUCCCAUUCUCAUUACUGCUUCUCCAAGCGAAUCUCUGUAGAGUUUCAGAGACUCGAACUGUGUUAUCAUUCCAUUCAAGUCCUCCGAUGAGGACUCCAACUGCAUUUUUGACAUCCUCAGCAGUAUGUCCUGGAAGAGAAGGCAAUGGUGAAAUUUCGCCAACAAUUGCUCCCUCUUCGGUGAAAGCCCUUAGCAAUAUUAGAGUCUCCAGCCGGUCAAAAAUCACACUGAAGUUCGCUUUCAGUAUGAUGUUCUUAUCCAUGAUCGCCUGGUCCAUUCUGAUACAAAGAGGGCCUGCCACUUUCUGCUUGGGUAUGAGCAUGGACCAGUCCCUUGACAUUUCCUCAAGAGUCAUGUCAGUUAGGUAACGCGACGCAGGUACAGAGGCCAUGGUCAUUUUAAGUGCCUCAUCGGAUUCUUCUUUCAGAAUCCGCUCCACUAUCUGCUUUCCAGCACGUGUGGCUGUCUUGAUGUCCAGACCGAGAGUACUGCCCCUUCCUCUUAGGGAUUUCUGAUCUCGGCGAAGCCGAUCAAGGAAUGGGGCAUCACCUAGUUCUUGGUCUGCAACUCGUUUGCGGACAUGCCAAAGAAAGCAAUCUACCUGAAAGCUUGACACAGUGUUUGGAUCCAUUAUGUUUUUGUCACCCUGCUUUUGCC |
| EPO | 5′-pppA | AGGAAAUAAGAGAGAAAAGAAGAGUAAGAAGAAAUAUAAGACCCCGGCGCCGCCACCAUGGGGGUCCAUGAGUGCCCAGCUUGGCUCUGGCUGCUUCUGUCUCUGUUGUCCCUGCCAUUGGGCCUGCCUGUACUGGGCGCACCACCUAGACUCAUAUGCGACAGCCGCGUCUUGGAACGAUAUCUCCUUGAGGCCAAGGAAGCAGAAAACAUCACCACCGGCUGCGCUGAGCAUUGUAGUCUGAAUGAAAACAUAACCGUUCCGGACACAAAGGUUAAUUUCUAUGCGUGGAAACGAAUGGAAGUAGGCCAGCAAGCUGUUGAGGUCUGGCAAGGGUUGGCACUUCUCUCAGAAGCUGUACUUCGCGGCCAGGCGCUCUUGGUCAACUCCAGUCAGCCAUGGGAGCCCCUCCAGCUCCAUGUUGAUAAGGCGGUAUCUGGUCUUCGAUCCCUGACGACUCUGCUUCGAGCGCUGGGGGCACAAAAGGAAGCCAUAUCUCCCCCUGAUGCGGCCUCUGCCGCGCCCCUCAGGACAAUCACAGCAGAUACUUUCAGAAAAUUGUUCAGGGUCUACUCCAAUUUCUUGCGGGGUAAGCUGAAGCUCUACACAGGCGAGGCAUGUCGAACGGGAGAUAGGUGAUAGG |
|  | 5′-pppG | GGGAAAUAAGAGAGAAAAGAAGAGUAAGAAGAAAUAUAAGACCCCGGCGCCGCCACCAUGGGGGUCCAUGAGUGCCCAGCUUGGCUCUGGCUGCUUCUGUCUCUGUUGUCCCUGCCAUUGGGCCUGCCUGUACUGGGCGCACCACCUAGACUCAUAUGCGACAGCCGCGUCUUGGAACGAUAUCUCCUUGAGGCCAAGGAAGCAGAAAACAUCACCACCGGCUGCGCUGAGCAUUGUAGUCUGAAUGAAAACAUAACCGUUCCGGACACAAAGGUUAAUUUCUAUGCGUGGAAACGAAUGGAAGUAGGCCAGCAAGCUGUUGAGGUCUGGCAAGGGUUGGCACUUCUCUCAGAAGCUGUACUUCGCGGCCAGGCGCUCUUGGUCAACUCCAGUCAGCCAUGGGAGCCCCUCCAGCUCCAUGUUGAUAAGGCGGUAUCUGGUCUUCGAUCCCUGACGACUCUGCUUCGAGCGCUGGGGGCACAAAAGGAAGCCAUAUCUCCCCCUGAUGCGGCCUCUGCCGCGCCCCUCAGGACAAUCACAGCAGAUACUUUCAGAAAAUUGUUCAGGGUCUACUCCAAUUUCUUGCGGGGUAAGCUGAAGCUCUACACAGGCGAGGCAUGUCGAACGGGAGAUAGGUGAUAGG |

**Supplementary Table S2.** Primers for IVT of RNAs. Yellow highlights T7 promoter, bold shows modified pair of the nucleotides.

| Transcript | RNA variant | Forward primer, 5′-3′ | Reverse primer, 5′-3′ |
| --- | --- | --- | --- |
| short viral | 5′-pppA | TAATACGACTCACTATT**A**GCAAAAGCAGGGTGACAA | **A**GCCAAAGAAAGCAATCTACCTG |
|  | 5′-pppG | TAATACGACTCACTATA**G**GCAAAAGCAGGGTGACAA | **G**GCCAAAGAAAGCAATCTACCTG |
| short viral antisense | antisense to  5′-pppA | AAGCTAATACGACTCACTATT**A**GCCAAAGAAAGCAATCTACC | **A**GCAAAAGCAGGGTGACAAAGAC |
|  | antisense to  5′-pppG | AAGCTAATACGACTCACTATA**G**GCCAAAGAAAGCAATCTACC | **G**GCAAAAGCAGGGTGACAAAGAC |
| Y5 WT | 5′-pppA | AAGCTAATACGACTCACTATT**A**GTTGGTCCGAGTGTTGTGGGTTAT | AAAACAGCA**A**GCTAGTCAAGCGCGGTTG |
|  | 5′-pppG | AAGCTAATACGACTCACTATA**G**GTTGGTCCGAGTGTTGTGGGTTATTG | AAAACAGCA**G**GCTAGTCAAGCGCGG |
| 78 nt Y5 antisense | antisense to 5′-pppA | AAGCTAATACGACTCACTATTGCA**A**GCTAGTCAAGCGCGG | **A**GTTGGTCCGAGTGTTGTGG |
|  | antisense to 5′-pppG | AAGCTAATACGACTCACTATAGCA**G**GCTAGTCAAGCGCGG | **G**GTTGGTCCGAGTGTTGTGG |
| Y5 AU clamp | 5′-pppA | AAGCTAATACGACTCACTATT**A**ATTAATCCGAGTGTTGTGGGTTATTGTT | AAAACAGCA**A**ATTAATCAAGCGCGGTTGTGGG |
|  | 5′-pppG | AAGCTAATACGACTCACTATA**G**ATTAATCCGAGTGTTGTGGGTTATTGTT | AAAACAGCA**G**ATTAATCAAGCGCGGTTG |
| Y5 GC clamp | 5′-pppA | AAGCTAATACGACTCACTATT**A**GCGGGGCCGAGTGTTGTGGGTTATTGTT | AAAACAGCA**A**GCGGGGCAAGCGCGGTTGTGGGGGGA |
|  | 5′-pppG | AAGCTAATACGACTCACTATA**G**GCGGGGCCGAGTGTTGTGGGTTATTGTT | AAAACAGCA**G**GCGGGGCAAGCGCGGTTGTGGGGGGA |
| Y5 WT (-) tail | 5′-pppA | AAGCTAATACGACTCACTATT**A**GTTGGTCCGAGTGTTGTGGGTTAT | **A**GCTAGTCAAGCGCGGTTGT |
|  | 5′-pppG | AAGCTAATACGACTCACTATA**G**GTTGGTCCGAGTGTTGTGGGTTAT | **G**GCTAGTCAAGCGCGGTTGTG |
| Y5 WT (-) tail antisense | antisense to  5′-pppA | AAGCTAATACGACTCACTATT**A**GCTAGTCAAGCGCGGTTGTG | **A**GTTGGTCCGAGTGTTGTGG |
| Y5 AU clamp (-) tail | 5′-pppA | AAGCTAATACGACTCACTATT**A**ATTAATCCGAGTGTTGTGGGTTATTGTT | **A**ATTAATCAAGCGCGGTTGTGGGG |
|  | 5′-pppG | AAGCTAATACGACTCACTATA**G**ATTAATCCGAGTGTTGTGGGTTATTGTT | **G**ATTAATCAAGCGCGGTTGTGGGG |
| Y5 GC clamp (-) tail | 5′-pppA | AAGCTAATACGACTCACTATT**A**GCGGGGCCGAGTGTTGTGGGTTATTGTT | **A**GCGGGGCAAGCGCGGTTGTGGGGGGA |
|  | 5′-pppG | AAGCTAATACGACTCACTATA**G**GCGGGGCCGAGTGTTGTGGGTTATTGTT | **G**GCGGGGCAAGCGCGGTTGTGGGGGGA |
| 72NT | 5′-pppA | GCGTAATACGACTCACTATT**A**GTAGAAACAAGGGTGTTTTTTAGT | **A**GCAAAAGCAGGGTGACAAAAACATAATG |
|  | 5′-pppG | GCGTAATACGACTCACTATA**G**GTAGAAACAAGGGTGTTTTTTAGT | **G**GCAAAAGCAGGGTGACAAAAACATAATG |
| 100NT | 5′-pppA | GCGTAATACGACTCACTATT**A**GTAGAAACAAGGGTGTTTTTTAGT | **A**GCAAAAGCAGGGTGACAAAAACATAATG |
|  | 5′-pppG | GCGTAATACGACTCACTATA**G**GTAGAAACAAGGGTGTTTTTTAGT | **G**GCAAAAGCAGGGTGACAAAAACATAATG |
| Y3 | 5′-pppA | AAGCTAATACGACTCACTATT**A**GCTGGTCCGAGTGCAGTGGTGTT | AAAA**A**GCTAGTCAAGTGAAGCAG |
|  | 5′-pppG | AAGCTAATACGACTCACTATA**G**GCTGGTCCGAGTGCAGTGGTGTT | AAAA**G**GCTAGTCAAGTGAAGCAG |
| Y4 | 5′-pppA | AAGCTAATACGACTCACTATT**A**GCTGGTCCGATGGTAGTGGGTTAT | AAAA**A**GCCAGTCAAATTTAGCAG |
|  | 5′-pppG | AAGCTAATACGACTCACTATA**G**GCTGGTCCGATGGTAGTGGGTTAT |  |
| tRNA | 5′-pppA | AAGCTAATACGACTCACTATT**A**GAGAGGCCTGGCCGAGTG | C**A**ACGAGGATGGGATTCGAACCCAC |
|  | 5′-pppG | AAGCTAATACGACTCACTATA**G**GAGAGGCCTGGCCGAGTG | C**G**ACGAGGATGGGATTCGAACCCAC |
| Seg. 8^th^ IAV | 5′-pppA | III class promoter TATA  GCGTAATACGACTCACTATA**A**GTAGAAACAAGGGTGTTTTTTAGT  II class promoter TATT  GCGTAATACGACTCACTATT**A**GTAGAAACAAGGGTGTTTTTTAGT | **A**GCAAAAGCAGGGTGACAAAAACATAATG |
|  | 5′-pppG | III class promoter TATA  GCGTAATACGACTCACTATA**G**GTAGAAACAAGGGTGTTTTTTAGT  II class promoter TATT  GCGTAATACGACTCACTATA**G**GTAGAAACAAGGGTGTTTTTTAGT | **G**GCAAAAGCAGGGTGACAAAAACATAATG |
| EPO | 5′-pppA | III class promoter TATA  TAATACGACTCACTATA**A**GGAAATAAGAGAGAAAAGAAGAG  II class promoter TATT  TAATACGACTCACTATT**A**GGAAATAAGAGAGAAAAGAAGAG | TGCCGCCCACTCAGAC |
|  | 5′-pppG | III class promoter TATA  TAATACGACTCACTATA**G**GGAAATAAGAGAGAAAAGAAGAG  II class promoter TATT  TAATACGACTCACTATT**G**GGAAATAAGAGAGAAAAGAAGAG | TGCCGCCCACTCAGAC |

**Supplementary Table S3.** Cell lines.

| Cell line | Source | Identifier |
| --- | --- | --- |
| Human HEK293 | ATCC | CRL-1573 |
| Human HEK293 RIG-I KO | CRISPR/Cas9 created | N/A |
| Human A549 | InvivoGen | a549d-nfis |
| Human A549 Dual RIG-I KO | InvivoGen | a549d-korigi |
| Human A549 Dual MDA-5 KO | InvivoGen | a549d-komda5 |
| Human A549 MAVS KO | Kindly provided by Prof. Tomasz Lipniacki | N/A |
| Human THP-1 | ATCC | TIB-202 |
| Human THP-1 Dual RIG-I KO | InvivoGen | thpd-korigi |
| Human THP-1 Dual MDA-5 KO | InvivoGen | thpd-komda5 |
| Human THP-1 Dual MAVS KO | InvivoGen | thpd-komavs |
| Human HEK-Blue IFN-α/β | InvivoGen | hkb-ifnab |
| Murine MEF | ATCC | CRL-2991 |
| Murine bone marrow derived macrophages | N/A | N/A |
| Primary murine fibroblasts | N/A | N/A |
| Murine B16-Blue IFN-α/β | InvivoGen | bb-ifnt1 |

**Supplementary Table S4.** Primary antibodies.

| Antigen | Vendor | Code | Dilution ratio |
| --- | --- | --- | --- |
| RIG-I | CellSignaling | 3743 | 1/1000 |
| pIRF3 | CellSignaling | 4947 | 1/1000 |
| IRF3 | Proteintech | 11312-1-AP | 1/1000 |
| MDA-5 | CellSignaling | 5321 | 1/1000 |
| MAVS | CellSignaling | 3993 | 1/1000 |
| α-tubulin | Proteintech | 11224-1-AP | 1/4000 |
| dsRNA | Jena Bioscience | 10010200 (J2) | 1/1000 |
